# Safety and immunogenicity of the two-dose heterologous Ad26.ZEBOV and MVA-BN-Filo Ebola vaccine regimen in children in Sierra Leone: a randomised, double-blind, controlled trial

**DOI:** 10.1016/S1473-3099(21)00128-6

**Published:** 2021-09-13

**Authors:** Muhammed O Afolabi, David Ishola, Daniela Manno, Babajide Keshinro, Viki Bockstal, Baimba Rogers, Kwabena Owusu-Kyei, Alimamy Serry-Bangura, Ibrahim Swaray, Brett Lowe, Dickens Kowuor, Frank Baiden, Thomas Mooney, Elizabeth Smout, Brian Köhn, Godfrey T Otieno, Morrison Jusu, Julie Foster, Mohamed Samai, Gibrilla Fadlu Deen, Heidi Larson, Shelley Lees, Neil Goldstein, Katherine E Gallagher, Auguste Gaddah, Dirk Heerwegh, Benoit Callendret, Kerstin Luhn, Cynthia Robinson, Brian Greenwood, Maarten Leyssen, Macaya Douoguih, Bailah Leigh, Deborah Watson-Jones

**Affiliations:** London School of Hygiene & Tropical Medicine, London, UK; EBOVAC Project, Kambia, Kambia district, Sierra Leone; London School of Hygiene & Tropical Medicine, London, UK; EBOVAC Project, Kambia, Kambia district, Sierra Leone; London School of Hygiene & Tropical Medicine, London, UK; Janssen Vaccines and Prevention BV, Leiden, Netherlands; Janssen Vaccines and Prevention BV, Leiden, Netherlands; EBOVAC Project, Kambia, Kambia district, Sierra Leone; College of Medicine and Allied Health Sciences, University of Sierra Leone, Freetown, Sierra Leone; London School of Hygiene & Tropical Medicine, London, UK; EBOVAC Project, Kambia, Kambia district, Sierra Leone; EBOVAC Project, Kambia, Kambia district, Sierra Leone; College of Medicine and Allied Health Sciences, University of Sierra Leone, Freetown, Sierra Leone; EBOVAC Project, Kambia, Kambia district, Sierra Leone; College of Medicine and Allied Health Sciences, University of Sierra Leone, Freetown, Sierra Leone; KEMRI-Wellcome Trust Research Programme, Kilifi, Kenya; Centre for Tropical Medicine and Global Health, University of Oxford, Oxford, UK; London School of Hygiene & Tropical Medicine, London, UK; EBOVAC Project, Kambia, Kambia district, Sierra Leone; London School of Hygiene & Tropical Medicine, London, UK; EBOVAC Project, Kambia, Kambia district, Sierra Leone; London School of Hygiene & Tropical Medicine, London, UK; EBOVAC Project, Kambia, Kambia district, Sierra Leone; London School of Hygiene & Tropical Medicine, London, UK; EBOVAC Project, Kambia, Kambia district, Sierra Leone; London School of Hygiene & Tropical Medicine, London, UK; EBOVAC Project, Kambia, Kambia district, Sierra Leone; London School of Hygiene & Tropical Medicine, London, UK; London School of Hygiene & Tropical Medicine, London, UK; EBOVAC Project, Kambia, Kambia district, Sierra Leone; College of Medicine and Allied Health Sciences, University of Sierra Leone, Freetown, Sierra Leone; London School of Hygiene & Tropical Medicine, London, UK; College of Medicine and Allied Health Sciences, University of Sierra Leone, Freetown, Sierra Leone; College of Medicine and Allied Health Sciences, University of Sierra Leone, Freetown, Sierra Leone; London School of Hygiene & Tropical Medicine, London, UK; Department of Health Metrics Sciences, University of Washington, Seattle, WA, USA; London School of Hygiene & Tropical Medicine, London, UK; Janssen Vaccines and Prevention BV, Leiden, Netherlands; London School of Hygiene & Tropical Medicine, London, UK; Janssen Research & Development, Beerse, Belgium; Janssen Research & Development, Beerse, Belgium; Janssen Vaccines and Prevention BV, Leiden, Netherlands; Janssen Vaccines and Prevention BV, Leiden, Netherlands; Janssen Vaccines and Prevention BV, Leiden, Netherlands; London School of Hygiene & Tropical Medicine, London, UK; Janssen Vaccines and Prevention BV, Leiden, Netherlands; Janssen Vaccines and Prevention BV, Leiden, Netherlands; College of Medicine and Allied Health Sciences, University of Sierra Leone, Freetown, Sierra Leone; London School of Hygiene & Tropical Medicine, London, UK; Mwanza Intervention Trials Unit, National Institute for Medical Research, Mwanza, Tanzania

## Abstract

**Background:**

Children account for a substantial proportion of cases and deaths from Ebola virus disease. We aimed to assess the safety and immunogenicity of a two-dose heterologous vaccine regimen, comprising the adenovirus type 26 vector-based vaccine encoding the Ebola virus glycoprotein (Ad26.ZEBOV) and the modified vaccinia Ankara vectorbased vaccine, encoding glycoproteins from the Ebola virus, Sudan virus, and Marburg virus, and the nucleoprotein from the Tai Forest virus (MVA-BN-Filo), in a paediatric population in Sierra Leone.

**Methods:**

This randomised, double-blind, controlled trial was done at three clinics in Kambia district, Sierra Leone. Healthy children and adolescents aged 1–17 years were enrolled in three age cohorts (12–17 years, 4–11 years, and 1–3 years) and randomly assigned (3:1), via computer-generated block randomisation (block size of eight), to receive an intramuscular injection of either Ad26.ZEBOV (5 × 10^10^ viral particles; first dose) followed by MVA-BN-Filo (1 × 10^8^ infectious units; second dose) on day 57 (Ebola vaccine group), or a single dose of meningococcal quadrivalent (serogroups A, C, W135, and Y) conjugate vaccine (MenACWY; first dose) followed by placebo (second dose) on day 57 (control group). Study team personnel (except for those with primary responsibility for study vaccine preparation), participants, and their parents or guardians were masked to study vaccine allocation. The primary outcome was safety, measured as the occurrence of solicited local and systemic adverse symptoms during 7 days after each vaccination, unsolicited systemic adverse events during 28 days after each vaccination, abnormal laboratory results during the study period, and serious adverse events or immediate reportable events throughout the study period. The secondary outcome was immunogenicity (humoral immune response), measured as the concentration of Ebola virus glycoprotein-specific binding antibodies at 21 days after the second dose. The primary outcome was assessed in all participants who had received at least one dose of study vaccine and had available reactogenicity data, and immunogenicity was assessed in all participants who had received both vaccinations within the protocol-defined time window, had at least one evaluable post-vaccination sample, and had no major protocol deviations that could have influenced the immune response. This study is registered at ClinicalTrials.gov, NCT02509494.

**Findings:**

From April 4, 2017, to July 5, 2018, 576 eligible children or adolescents (192 in each of the three age cohorts) were enrolled and randomly assigned. The most common solicited local adverse event during the 7 days after the first and second dose was injection-site pain in all age groups, with frequencies ranging from 0% (none of 48) of children aged 1–3 years after placebo injection to 21% (30 of 144) of children aged 4–11 years after Ad26.ZEBOV vaccination. The most frequently observed solicited systemic adverse event during the 7 days was headache in the 12–17 years and 4–11 years age cohorts after the first and second dose, and pyrexia in the 1–3 years age cohort after the first and second dose. The most frequent unsolicited adverse event after the first and second dose vaccinations was malaria in all age cohorts, irrespective of the vaccine types. Following vaccination with MenACWY, severe thrombocytopaenia was observed in one participant aged 3 years. No other clinically significant laboratory abnormalities were observed in other study participants, and no serious adverse events related to the Ebola vaccine regimen were reported. There were no treatment-related deaths. Ebola virus glycoprotein-specific binding antibody responses at 21 days after the second dose of the Ebola virus vaccine regimen were observed in 131 (98%) of 134 children aged 12–17 years (9929 ELISA units [EU]/mL [95% CI 8172–12 064]), in 119 (99%) of 120 aged 4–11 years (10 212 EU/mL [8419–12 388]), and in 118 (98%) of 121 aged 1–3 years (22 568 EU/mL [18 426–27 642]).

**Interpretation:**

The Ad26.ZEBOV and MVA-BN-Filo Ebola vaccine regimen was well tolerated with no safety concerns in children aged 1–17 years, and induced robust humoral immune responses, suggesting suitability of this regimen for Ebola virus disease prophylaxis in children.

**Funding:**

Innovative Medicines Initiative 2 Joint Undertaking and Janssen Vaccines & Prevention BV.

## Introduction

In the 2014–16 outbreak of Ebola virus disease in west Africa that resulted in 28 652 cases and 11 325 deaths,^1,2^ approximately 20% of cases were in children younger than 15 years.^3,4^ Similarly, in the 2018–20 Ebola virus disease outbreak in the Democratic Republic of the Congo, approximately 30% of Ebola virus disease cases were in children younger than 18 years.^5^ Children, especially those younger than 5 years, have a more rapid clinical progression and a higher risk of death than adults.^3^ These features underscore the need for an effective Ebola prevention strategy in paediatric populations.

The clinical evaluation of several candidate vaccines was accelerated because of both outbreaks.^6^ A live-attenuated, single-dose, recombinant vesicular stomatitis virus-vectored vaccine expressing the Ebola virus glycoprotein (rVSV-ZEBOV-GP) was shown to provide protection against Ebola virus disease during the 2014–16 Ebola outbreak in Guinea by use of a ring vaccination approach.^7^ As part of the outbreak response, this vaccine was also used in adults and in children aged 1–17 years during the 2018–20 outbreak in DR Congo, under expanded access.^8^ rVSV-ZEBOV-GP has received conditional approval by the European Medicines Agency (EMA)^9^ and the US Food and Drug Administration (FDA) for use in adults.^10^ Following recommendations on vaccination against Ebola virus disease by the Strategic Advisory Group of Experts on Immunization,^11^ a two-dose Ebola vaccine regimen, comprising the adenovirus type 26 (Ad26) vector-based vaccine encoding the Ebola virus glycoprotein (Ad26.ZEBOV) and the modified vaccinia Ankara (MVA) vector-based vaccine, encoding glycoproteins from the Ebola virus, Sudan virus, and Marburg virus, and the nucleoprotein from the Tai Forest virus (MVA-BN-Filo), has also been used to vaccinate adults and children aged 1–17 years in DR Congo and Rwanda. In July 2020, the European Commission granted approval of this two-dose heterologous vaccine regimen for use in children and adults under exceptional circumstances.^12^

The Ad26.ZEBOV and MVA-BN-Filo vaccine regimen was evaluated in a randomised controlled trial in a community affected by Ebola during the epidemic in west Africa.^13^ This vaccine regimen has been shown to have an acceptable safety profile and to induce robust humoral immune responses in adults.^13^ We aimed to assess the safety and immunogenicity of this vaccine regimen in children aged 1–17 years from the same community. The data presented in this report contributed to the European Commission’s approval for use of the vaccine regimen in adults and children.^12^

## Methods

### Study design and participants

This randomised, double-blind, controlled trial was done at three clinics in Kambia district, located in the north western province of Sierra Leone, an area that was severely affected by the 2014–16 Ebola virus disease outbreak. Eligible participants were healthy children or adolescents aged 1–17 years, who were enrolled in three age cohorts (12–17 years, 4–11 years, and 1–3 years). An independent data monitoring committee assessed the safety results of each age cohort before proceeding with the enrolment of the first 96 participants in the next cohort. This trial also enrolled healthy adults (aged ≥18 years), and data from this cohort are presented in a separate publication.^13^

Community engagement activities, including meetings, radio discussion programmes, and drama group role-plays, were held in the local community to provide information about the trial and discuss any questions or concerns that parents or guardians had. Parents or guardians of apparently healthy children aged 1–17 years who expressed an interest in the trial were subsequently invited, together with their children, to the trial clinics for eligibility assessments. A trial physician obtained a detailed medical history from the parents or guardians of a potentially eligible child and did a physical examination to ascertain whether the child was healthy. A blood sample was then taken for measurement of baseline haematological and biochemical variables. To exclude the possibility of pregnancy, urinary β-human chorionic gonadotropin tests were done in female participants considered to have childbearing potential. The full list of inclusion and exclusion criteria is presented in the trial protocol (appendix pp 85–89). Before enrolment, parents or guardians were given information about the trial in a language they understood and, after passing a test of understanding, they provided written informed consent for their child to join the trial. Children aged 7 years and older also gave written assent.

The trial protocol was approved by the Sierra Leone Ethics and Scientific Review Committee, the London School of Hygiene & Tropical Medicine Ethics Committee, and the Pharmacy Board of Sierra Leone. The trial was done according to the International Council for Harmonisation of Technical Requirements for Pharmaceuticals for Human Use Good Clinical Practice guidelines, and was monitored by ICON Government and Public Health Solutions (an external contract research organisation). The study protocol is included in the appendix (pp 26–155).

### Randomisation and masking

Study participants were randomly assigned (3:1) to receive either the Ad26.ZEBOV and MVA-BN-Filo vaccine regimen (Ebola vaccine group) or the meningococcal quadrivalent (serogroups A, C, W135, and Y) conjugate vaccine (MenACWY) and placebo (control group). Randomisation was done centrally by use of a computer-generated block randomisation (block size of eight) schedule via an interactive web-response system (IWRS), operated by a study pharmacist. Study participants, their parents or guardians, and all study team members (except for study pharmacists who operated the IWRS) were masked to study vaccine allocation. The dispensing syringes containing the treatment allocated to each participant all contained the same volume and were taped to conceal the colour of the liquid inside. This process guaranteed treatment concealment until after completion of study follow-up visits by all participants.

### Procedures

Participants in the Ebola vaccine group received Ad26.ZEBOV (5 × 10^10^ viral particles; first dose) followed by MVA-BN-Filo (1 × 10^8^ infectious units; second dose) 56 days after the first dose, and those in the control group received 0·5 mL MenACWY (first dose), followed by 0·5 mL saline (sodium chloride solution [0.9%]; second dose) 56 days after the first dose. Study participants aged younger than 2 years at enrolment also received a third vaccination of MenACWY at 3 months after the second dose. The study vaccines were administered intramuscularly into the deltoid muscle for participants aged 4–17 years, and intramuscularly into the anterolateral thigh in participants aged 1–3 years.

As previously described,^14,15^ Ad26.ZEBOV was manufactured by Janssen Vaccines & Prevention BV (Leiden, Netherlands) and MVA-BN-Filo by Bavarian Nordic (Kvistgaard, Denmark) under good manufacturing practice conditions. The MenACWY vaccine (Menveo [GSK Vaccines, Brentford, UK]; or Nimenrix [Pfizer, New York, NY, USA) was chosen as a comparator vaccine to provide some benefits to children in the control group; bacterial meningitis is endemic in the study area, and the meningitis vaccine is not included in the routine childhood immunisation schedule in Sierra Leone.

After each vaccination, study participants were directly observed in the trial clinics for 30 min and then followed up at home. Trained field assistants visited the study participants at home each day for 7 days after each vaccination to administer a standardised, purpose-designed reactogenicity diary card to the study participant, their parent or guardian, or both. Assessments of all participants for solicited, unsolicited, local and systemic adverse events during the 28-day follow-up after each vaccination were done in two stages. During the first 7 days after each vaccination, a trained field assistant visited a study participant at home to collect data on local and systemic adverse events using a purpose-designed diary card. From day 8 onwards, assessments for adverse events were done at the trial clinics by a study physician using a standardised case report form.

Blood samples for laboratory safety assessments were collected on day 8 after each vaccination and on day 57 before vaccination with the second dose, to assess complete blood count, and alanine aminotransferase, aspartate aminotransferase, and serum creatinine concentrations.

All participants were followed up for serious adverse events and immediate reportable adverse events throughout the study. Parents or guardians were provided a 24-h telephone number to contact a study physician during the study period. All participants were followed up for safety for up to 12 months after the first dose. Any neuroinflammatory disorders categorised as immediate reportable events (listed in appendix p 122) were reported to the trial sponsor (Janssen Vaccines and Prevention BV) within 24 h. A study physician graded every adverse event by intensity and judged for relatedness to the study. Grade 1 (mild) adverse events were those that were tolerated easily, causing minimal or no interference with usual social and functional activities. Grade 2 (moderate) adverse events were those causing greater than minimal interference with usual social and functional activities. Grade 3 (severe) adverse events prevented usual social and functional activities.

Ebola virus glycoprotein-specific binding antibody responses were measured by the Ebola virus glycoprotein Filovirus Animal Non-Clinical Group ELISA (validated by and done at Q^2^ Solutions Vaccine Testing Laboratory (San Juan Capistrano, CA, USA) immediately before the first and second doses, at 21 days after the second dose, 6 months after the second dose, and 1 year after the first dose. In a randomly selected subset of participants from each age cohort, Ebola virus glycoprotein-specific neutralising antibody responses were assessed by use of an Ebola virus glycoprotein (Makona strain) pseudovirion neutralisation assay, which was developed and validated by Monogram Biosciences (San Francisco, CA, USA), where this analysis was done. Neutralising antibody responses were assessed immediately before the first dose, at 21 days after the second dose and at 1 year after the first dose. The presence of neutralising antibodies against the Ad26 vector backbone was measured at baseline by use of an Ad26-specific virus neutralisation assay, which was developed and qualified by Janssen Vaccines & Prevention BV, where this analysis was done. The presence of neutralising antibodies against the MVA vector backbone was measured at baseline by use of a plaque reduction neutralisation test, which was developed and validated by Bavarian Nordic (Planegg, Germany), where this analysis was done.

### Outcomes

The primary endpoint was safety, measured as the occurrence of: (1) solicited local and systemic adverse events during a 7-day follow-up period after each vaccination (starting from day 1 for the first dose and from day 57 for the second dose), (2) unsolicited systemic adverse events during a 28-day follow-up period after each vaccination, (3) abnormal laboratory results during the study period; and (4) serious adverse events or immediate reportable events throughout the study period. The toxicity scales used for the assessments of clinical laboratory values were based on the US FDA Toxicity Grading Scale for Healthy Adults and Adolescent Volunteers Enrolled in Preventive Vaccine Clinical Trials 2 and the Division of Microbiology and Infectious Diseases Toxicity Tables for use in trials enrolling children older than 3 months.^16^

The secondary endpoint was immunogenicity, measured by the Ebola virus glycoprotein-specific binding antibody concentrations at 21 days after the second dose. Responders were defined as those with either a negative ELISA result at baseline and a positive post-baseline value of more than 2·5 times the lower limit of quantification (LLOQ; 36·11 ELISA units [EU]/mL), or a positive result at baseline with a post baseline value that was 2.5-times higher than the baseline value. The exploratory outcomes were to assess Ebola virus glycoprotein-specific binding antibody concentrations at baseline, day 57, and day 360, and neutralising antibody titres directed against Ebola virus glycoprotein or the Ad26 and MVA vectors. Participants were considered as responders for the pseudovirion neutralisation assay if a sample was negative at baseline and positive post-baseline and the post-baseline value was greater than two times the LLOQ (a half maximal inhibitory concentration [IC_50_] titre of 120), or samples were positive both at baseline and post-baseline and there was a greater than two-times increase from baseline. Participants were considered as positive for the Ad26-specific virus neutralisation assay if a sample was greater than the LLOQ (a 90% inhibitory concentration [IC_90_] titre of 17), and positive for the plaque reduction neutralisation test if the sample was greater than the LLOQ (an IC_50_ titre of 8). Only data from baseline samples are presented.

### Statistical analysis

A sample size of 192 children (144 in the Ebola vaccine group and 48 in the control group) in each age cohort was calculated to provide a probability of 99% or higher of observing at least one serious adverse event in the Ebola vaccine group, if the true incidence of the serious adverse event is 10% or higher in each age cohort. Subsets of participants were selected for the analysis in the Ebola virus glycoprotein pseudovirion neutralisation assay and MVA plaque reduction neutralisation test. These subsets were not based on a separate sample size calculation, but were instead based on the number of samples that could be analysed in a reasonable amount of time, and considered large enough to provide a representative characterisation of the immune responses. For the analysis of the Ebola virus glycoprotein-specific neutralising antibody response, a subset of 165 participants (54 [28%] of 191 participants in the 12–17 years cohort, 55 [29%] of 192 in the 4–11 years cohort, and 56 [29%] of 192 in the 1–3 years cohort) were selected at random with SAS (version 9.2) in a 3:1 ratio of Ebola vaccine group participants to control group participants to ensure that the distribution of the selected participants was similar to the overall distribution of participants across the study groups. Random selection was done before the analysis of the samples among 534 participants with available samples and no protocol deviations that could have influenced the immune response. The neutralising antibody response against the Ad26 vector backbone was measured at baseline (immediately before the first dose) in 188 (98%) of 191 participants in the 12–17 years cohort, 179 (93%) of 192 in the 4–11 years cohort, and 164 (85%) of 192 in the 1–3 years cohort. For the analysis of neutralising antibody responses against the MVA vector, 166 participants (55 [29%] of 191 in the 12–17 years cohort, 55 [29%] of 192 in the 4–11 years cohort, and 56 [29%] of 192 in the 1-3 years cohort) were selected at random using the same methods as used for selection of participants for measuring Ebola virus glycoprotein-specific neutralising antibody responses.

All statistical analyses were done using SAS version 9.2. Descriptive analysis was done without formal hypothesis testing, and the results are presented by vaccination group. The full analysis set for safety comprised all participants who received at least one dose of study vaccine and had available reactogenicity data. The analysis set for immunogenicity (per-protocol) included all vaccinated children who received both vaccinations within the protocol-defined time window, had at least one evaluable post-vaccination immunogenicity sample, and had no major protocol deviations that could have influenced the immune response.

Binding antibody responses against Ebola virus glycoprotein are shown as geometric mean concentrations (GMCs), and neutralising antibody activity is shown as geometric mean titres (GMTs), both with their associated 95% CIs. All values less than the LLOQ were imputed as half the LLOQ value. We calculated Spearman’s correlation coefficients, correcting for age cohort (ie, partial correlation), to assess the association between Ebola virus glycoprotein-specific binding antibodies and pseudovirion neutralisation assay titres at 21 days after the second dose.

Post-hoc analyses were done to evaluate any potential influence of Ebola virus glycoprotein-specific binding antibodies present at baseline on Ebola virus glycoprotein-specific binding antibody concentrations at 21 days after the second dose (ie, by stratifying binding antibody concentrations at 21 days after the second dose by binding antibody concentrations at baseline, and by a Spearman’s correlation coefficient analysis).

This study is registered with ClinicalTrials.gov, NCT02509494.

### Role of the funding source

The Innovative Medicines Initiative 2 Joint Undertaking had no role in study design, data collection, data analysis, data interpretation, or writing of this report. Janssen Vaccines & Prevention BV had a role in study design, data collection, data analysis, data interpretation, and writing of the report.

## Results

**Figure 1:**
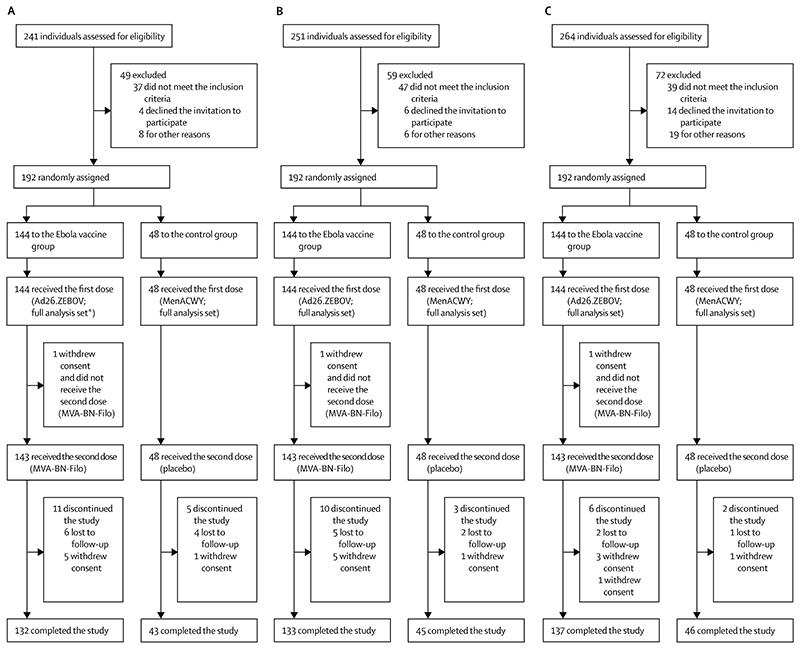
Trial profiles for the 12–17 years (A), 4–11 years (B), and 1–3 years (C) age cohorts Ad26.ZEBOV=adenovirus type 26 vector-based vaccine encoding the Ebola virus glycoprotein. MenACWY=meningococcal quadrivalent (serogroups A, C, W135, and Y) conjugate vaccine. MVA-BN-Filo=modified vaccinia Ankara vector-based vaccine, encoding glycoproteins from the Ebola virus, Sudan virus, and Marburg virus, and the nucleoprotein from the Tai Forest virus. *One participant received Ad26.ZEBOV followed by placebo and was therefore excluded from further analyses.

From April 4, 2017, to July 5, 2018, 576 (192 in each age cohort) were enrolled and randomly assigned. The total numbers of children screened in each age cohort and the numbers excluded and why are shown for each age cohort in figure 1. Baseline demographic characteristics of participants in each age cohort are summarised (table).

**Figure 2:**
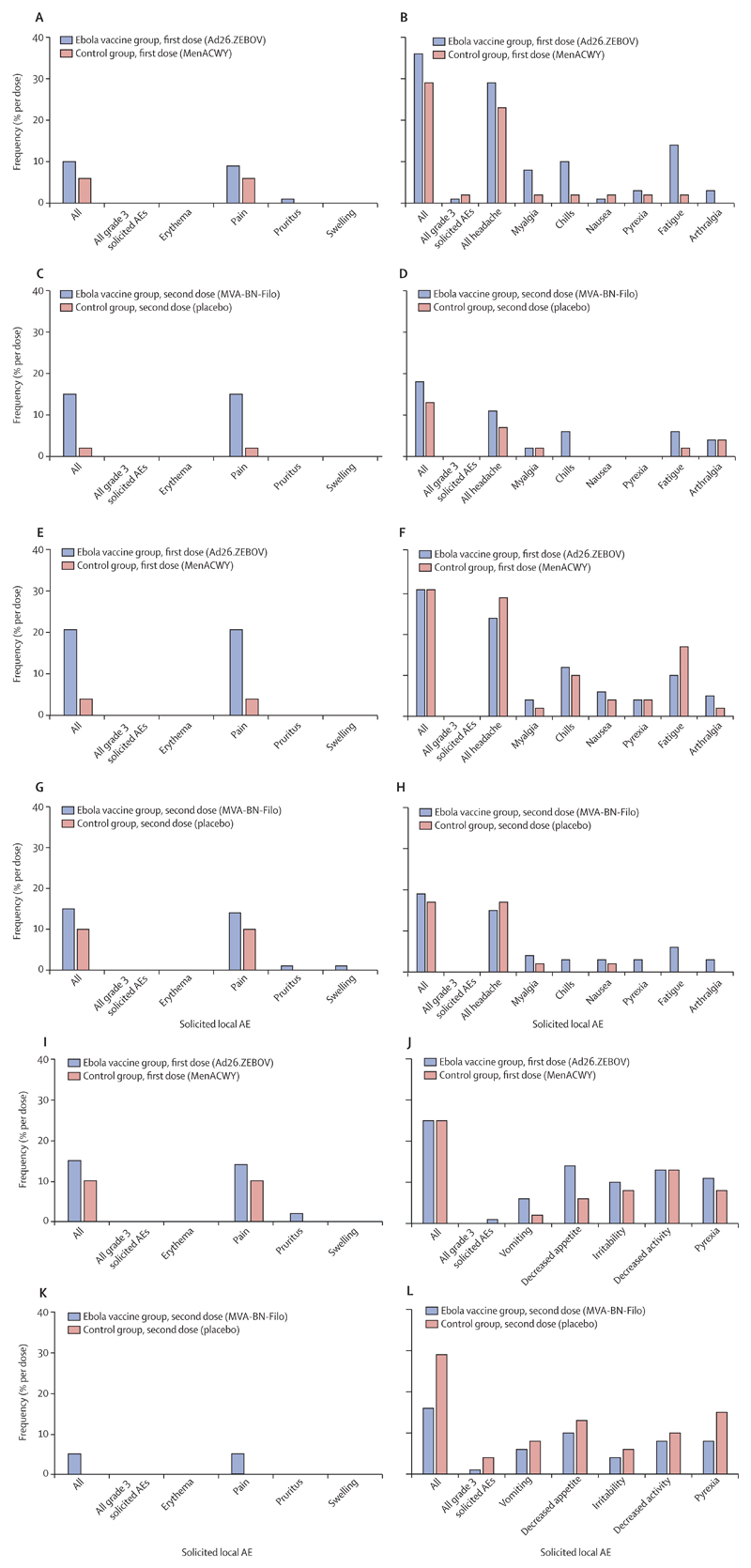
Solicited local and systemic AEs in the paediatric cohorts Solicited local (A) and systemic (B) AEs during 7 days after the first dose, and solicited local (C) and systemic (D) AEs during 7 days after the second dose in the 12–17 years age cohort. Solicited local (E) and systemic (F) AEs during 7 days after the first dose, and solicited local (G) and systemic (H) AEs during 7 days after the second dose in the 4–11 years age cohort. Solicited local (I) and systemic (J) AEs during 7 days after the first dose, and solicited local (K) and systemic (L) AEs during 7 days after the second dose in the 1–3 years age cohort. Ad26.ZEBOV=adenovirus type 26 vector-based vaccine encoding the Ebola virus glycoprotein. AE=adverse event. MenACWY=meningococcal quadrivalent (serogroups A, C, W135, and Y) conjugate vaccine. MVA-BN-Filo=modified vaccinia Ankara vector-based vaccine, encoding glycoproteins from the Ebola virus, Sudan virus, and Marburg virus, and the nucleoprotein from the Tai Forest virus.

Overall, solicited adverse events were mostly mild to moderate (grade 1 and 2) in severity (figure 2; appendix p 5). In all age cohorts, the most frequent solicited local adverse event was injection-site pain after any vaccination (figure 2; appendix p 6). No grade 3 solicited local adverse events were observed after any vaccination in any age cohort. In the 12–17 years age cohort, solicited local adverse events were reported in 14 (10%) of 143 Ebola vaccine recipients and in three (6%) of 48 MenACWY vaccine recipients at 7 days after the first dose and in 21 (15%) of 142 Ebola vaccine recipients and one (2%) of 46 placebo recipients at 7 days after the second dose (figure 2A, C). In the 4–11 years age cohort, at least one solicited local adverse event was reported in 30 (21%) of 144 Ebola vaccine recipients and two (4%) of 48 MenACWY vaccine recipients at 7 days after the first dose and in 22 (15%) of 144 Ebola vaccine recipients and five (10%) of 48 placebo recipients at 7 days after the second dose (figure 2E, G; appendix p 6). In the 1–3 years age cohort, at least one solicited local adverse event was observed in 21 (15%) of 144 children in the Ebola vaccine group and five (10%) of 48 in the control group at 7 days after the first dose and in seven (5%) of 143 in the Ebola vaccine group and none in the control group at 7 days after the second dose (figure 2I, K; appendix p 6).

Error bars show the 95% CIs. The black dotted line represents the LLOQ. Day 1 is baseline, day 57 is 56 days after the first dose, day 78 is 21 days after the second dose, day 240 is 179 days after the second dose, and day 360 is 359 days after the first dose. Ad26.ZEBOV=adenovirus type 26 vector-based vaccine encoding the Ebola virus glycoprotein. EU=ELISA units. LLOQ=lower limit of quantification. MenACWY=meningococcal quadrivalent (serogroups A, C, W135, and Y) conjugate vaccine. MVA-BN-Filo=modified vaccinia Ankara vector-based vaccine, encoding glycoproteins from the Ebola virus, Sudan virus, and Marburg virus, and the nucleoprotein from the Tai Forest virus.

Solicited systemic adverse events occurred at a higher frequency than solicited local adverse events, ranging from 13% (six of 46) after placebo injection to 36% (52 of 143) after Ad26.ZEBOV vaccination in children aged 12–17 years, 17% (eight of 48) after placebo injection to 31% after Ad26.ZEBOV (45 of 144) or MenACWY (15 of 48) vaccination in children aged 4–11 years, and 16% (23 of 143) after MVA-BN-Filo vaccination and 29% (14 of 48) after placebo injection in children aged 1–3 years (figure 2; appendix pp 7–8). Headache, fatigue, and chills were the most frequently reported solicited systemic adverse events after any vaccination in the 12–17 years and 4–11 years age cohorts (figures 2B, D, F, and H; appendix pp 7–8), whereas pyrexia, decreased appetite, and decreased activity were the most frequently observed solicited systemic adverse events in the 1–3 years age cohort (figure 2J, L; appendix pp 7–8). The frequency of pyrexia was higher in the 1–3 years age cohort than in the other age cohorts, regardless of the vaccine given (appendix pp 7–8). Grade 3 solicited systemic adverse events were infrequently observed after any vaccine in all age cohorts.

The most frequent unsolicited adverse event after the first and second doses was malaria in all age cohorts, irrespective of the type of vaccine given (appendix p 9). None of the adverse events were considered as related to the study vaccine. Grade 3 unsolicited adverse events were infrequently observed after vaccination, regardless of the type of vaccine given (appendix p 9). In the 12–17 years age cohort, 16 (8%) of 191 participants had at least one grade 3 unsolicited adverse event: five (3%) of 143 after the Ad26.ZEBOV vaccine, four (3%) of 142 after the MVA-BN-Filo vaccine, four (8%) of 48 after the MenACWY vaccine, and three (7%) of 46 after the placebo injection. The most frequent grade 3 unsolicited adverse event in this age cohort was decreased haemoglobin concentrations, which was observed in three (2%) participants after the Ad26.ZEBOV vaccine, four (3%) after the MVA-BN-Filo vaccine, two (4%) after the MenACWY vaccine, and three (7%) after the placebo injection. In two (2%) participants after the MVA-BN-Filo vaccine and one (2%) after the MenACWY vaccine the grade 3 haemoglobin event was considered to be related to the study vaccine. No grade 3 unsolicited adverse events were observed in the 4–11 years age cohort after the first or second doses. 17 (9%) of 192 participants in the 1–3 years age cohort had at least one grade 3 unsolicited adverse event: five (3%) of 144 after the Ad26. ZEBOV vaccine, ten (7%) of 143 after the MVA-BN-Filo vaccine, and two (4%) of 48 after the placebo injection. The most frequent grade 3 unsolicited adverse event in this age cohort was anaemia, which was reported in one (1%) participant after the Ad26.ZEBOV vaccine and in six (4%) participants after the MVA-BN-Filo vaccine.

No serious adverse events or deaths related to the Ebola vaccine regimen were observed during the study period. A total of 49 serious adverse events were reported in 24 participants (appendix p 10). Apart from one case of acute severe asthma in a participant aged 4–11 years in the Ebola vaccine group, all serious adverse events were related to infectious diseases or complications of malaria (anaemia, iron deficiency anaemia, thrombocytopenia, and febrile convulsion). 33 (67%) of 49 serious adverse events were observed after the first 28 days following any vaccination. One serious adverse event (severe thrombocytopenia), observed in a participant aged 3 years approximately 50 days after receiving the MenACWY vaccine, was considered to be possibly related to this vaccine, and was reported as a suspected unexpected serious adverse reaction. Two serious adverse events leading to death were recorded: one participant aged 17 years in the control group died of severe typhoid fever on day 319, and one participant aged 3 years in the Ebola vaccine group died of severe malaria and severe anaemia on day 74, 22 days after receiving the second dose. Both deaths were considered as unrelated to the study vaccine.

According to the US FDA Toxicity Grading Scale,^16^ a grade 3 change in haemoglobin concentrations from baseline was observed in two (1%) of 142 participants in the 12–17 years age cohort within the first 28 days after Ad26.ZEBOV vaccination, in eight (6%) of 141 after MVA-BN-Filo vaccination, in two (4%) of 48 after MenACWY vaccination, and in three (7%) of 46 after placebo injection. No other grade 3 laboratory abnormalities were observed in this age cohort (appendix pp 11–13). All participants in the 12–17 years age cohort who met the US FDA criteria for a grade 3 change in haemoglobin concentrations frombaseline had a haemoglobin value within the adapted normal laboratory ranges for the region.^17^ The change from baseline grading scale parameter for haemoglobin only applied to the 12–17 years age cohort, whereas grading of haemoglobin concentrations in the two younger age cohorts was based on the absolute value. In the 4–11 years age cohort, all grade 3 laboratory abnormalities were observed in one (1%) of 144 participants, at most. In the 1–3 years age cohort, all grade 3 laboratory abnormalities were observed in three (2%) of 144 participants at most, except for grade 3 haemoglobin values, which were observed in one (1%) of 144 participants after Ad26. ZEBOV vaccination, six (4%) of 143 after MVA-BN-Filo vaccination, and none after MenACWY vaccination or placebo injection (appendix pp 11–13). No immediate reportable events were observed.

**Figure 3:**
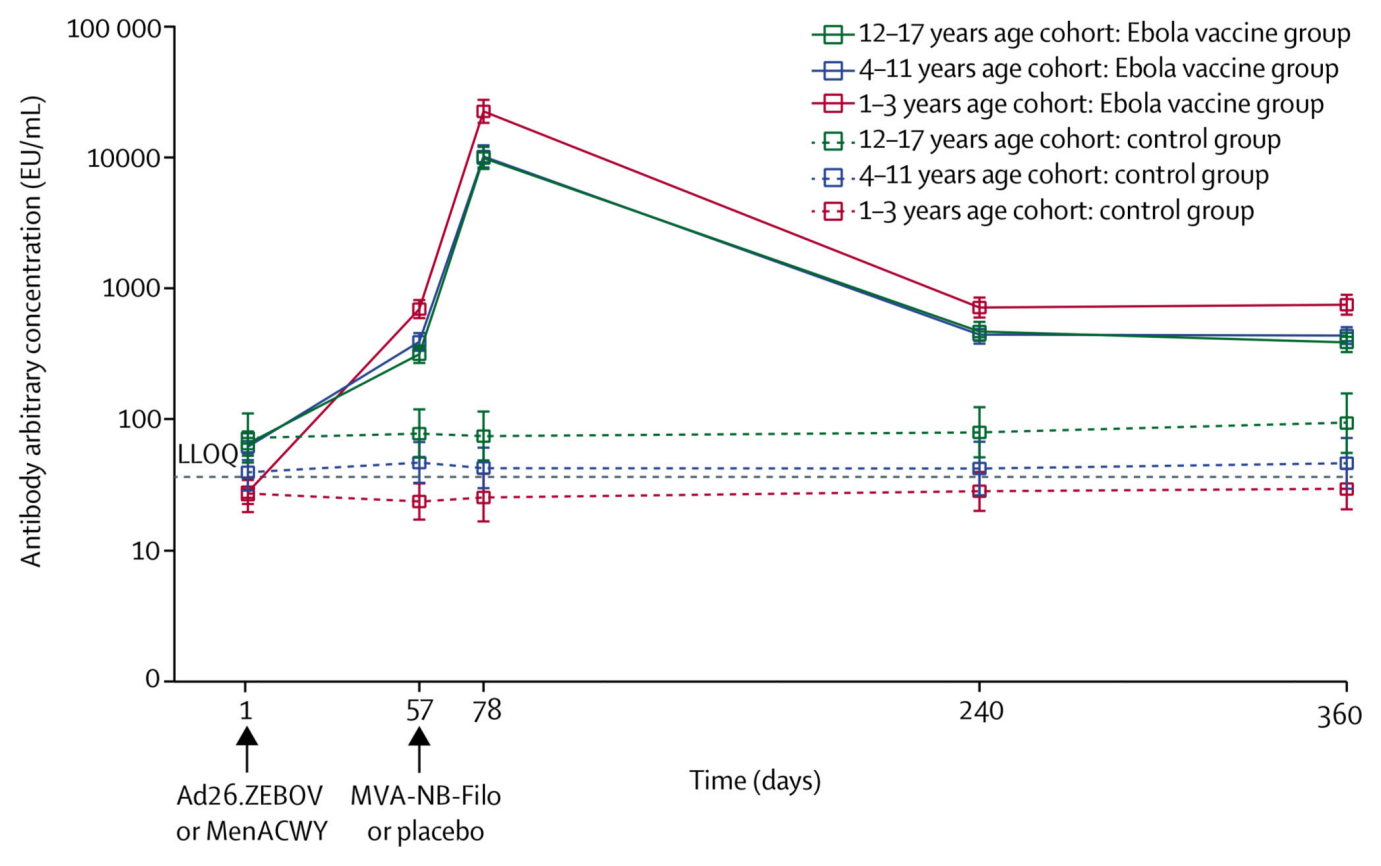
Geometric mean concentrations of Ebola virus glycoprotein-specific binding antibodies before and after each vaccination

Ebola virus glycoprotein-specific binding antibody results are summarised in figure 3 and in the appendix (pp 14–15). At 21 days after the second dose (day 78), Ebola virus glycoprotein-specific binding antibody responses in the Ebola vaccine group were observed in 118 (98%) of 121 participants in the 1–3 years age cohort (GMC 22 568 EU/mL, 95% CI 18 426–27 642), 119 (99%) of 120 in the 4–11 years age cohort (10 212 EU/mL, 8419–12 388), and 131 (98%) of 134 in the 12–17 years age cohort (9929 EU/mL, 8172–12 064; figure 3; appendix pp 14–15). Before administration of MVA-BN-Filo, both the GMC and proportion of responders were higher in the 1–3 years age cohort (115 [94%] of 122 participants; GMC 693 EU/mL [95% CI 591–812]) than in the 12–17 years age cohort (91 [64%] of 142; 314 EU/mL [269–366]) or the 4–11 years cohort (92 [71%] of 129; 390 EU/mL [334–456]). Compared with 21 days after the second dose (day 78), Ebola virus glycoprotein-specific binding antibody concentrations were lower on day 240 (6 months after the second dose) in all age cohorts, but responses persisted in 99 (73%) of 135 participants in the 12–17 years age cohort, 90 (74%) of 122 in the 4–11 years age cohort, and 111 (93%) of 119 in the 1–3 years age cohort (appendix pp 14–15). GMCs remained stable between day 240 and day 360 (1 year after the first dose), with Ebola virus glycoprotein-specific binding antibody responses observed in 92 (70%) of 132 participants in the 12–17 years age cohort, 85 (71%) of 119 in the 4–11 years age cohort, and 112 (96%) of 117 in the 1–3 years age cohort on day 360 (appendix pp 14–15).

**Figure 4:**
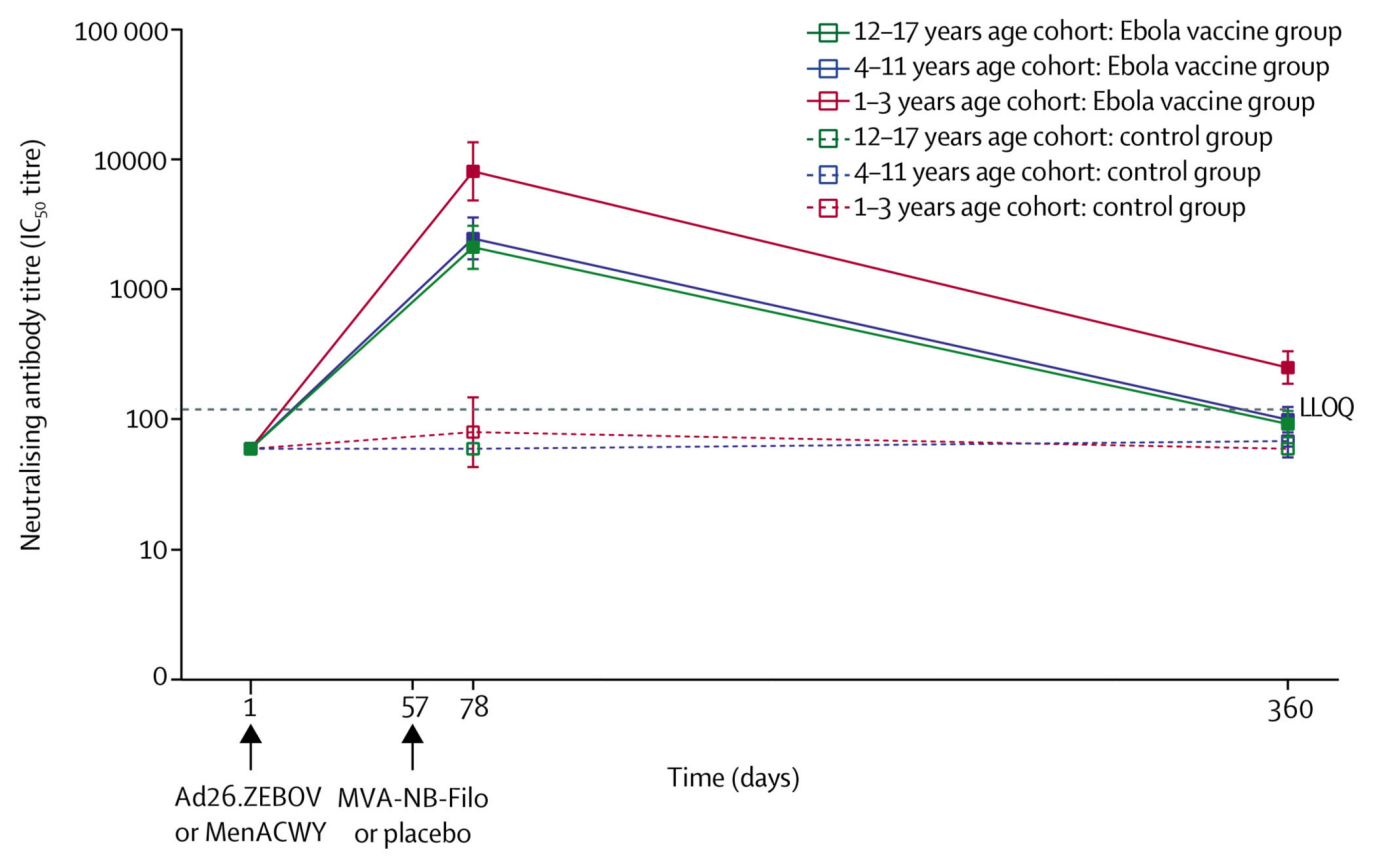
Ebola virus glycoprotein-specific neutralising antibody responses before and after each vaccination The response profile for each group is shown as geometric mean titres, measured by use of the pseudovirion neutralisation assay. The error bars show the 95% CIs. The black dotted line represents the LLOQ. Day 1 is baseline, day 57 is 56 days after the first dose, day 78 is 21 days after the second dose, day 240 is 179 days after the second dose, and day 360 is 359 days after the first dose. Ad26.ZEBOV=adenovirus type 26 vector-based vaccine encoding the Ebola virus glycoprotein. IC_50_=half maximal inhibitory concentration. LLOQ=lower limit of quantification. MenACWY=meningococcal quadrivalent (serogroups A, C, W135, and Y) conjugate vaccine. MVA-BN-Filo=modified vaccinia Ankara vector-based vaccine, encoding glycoproteins from the Ebola virus, Sudan virus, and Marburg virus, and the nucleoprotein from the Tai Forest virus.

At 21 days after the second dose (day 78), Ebola virus glycoprotein-specific neutralising antibody responses were detected in 94–95% of participants in the Ebola vaccine group across the three age cohorts (figure 4; appendix pp 16–17). The GMT in participants in the 1–3 years age cohort (IC_50_ titre 8142 [95% CI 4869–13 615]) was three to four times higher than the GMT in participants in the 12–17 years (2120 [1444–3111]) and the 4–11 years (2483 [1719–3587]) age cohorts (figure 4; appendix pp 16–17). At 21 days after the second dose, there was a strong positive correlation (corrected for age cohort) between Ebola virus glycoprotein-specific neutralising antibody and Ebola virus glycoprotein-specific binding antibody concentrations (partial *r*=0·881; appendix p 19). On day 360 (1 year after the first dose), neutralising antibody responses were observed in three (8%) of 40 participants in the 12–17 years age cohort, six (15%) of 40 in the 4–-11 years age cohort, and 18 (49%) of 37 in the 1–3 years age cohort (appendix pp 16–17). The GMT value was either low (IC_50_ titre 252 [95% CI 189–336] in participants in the 1–3 years age cohort) or less than the LLOQ (in children aged 4–17 years) on day 360.

Neutralising antibodies against the Ad26 vector backbone in the Ebola vaccine group were observed in 111 (78%) of 142 participants aged 12–17 years (IC_90_ GMT 77 [95% CI 58–101]), 103 (77%) of 134 aged 4–11 years (143 [101–201]), and 25 (20%) of 124 aged 1–3 years (19 [<LLOQ–26]; appendix p 18). Similar results were observed in the control group. In post-hoc analyses corrected for age cohort effect, a negligible negative correlation was observed between baseline Ad26-specific titres and vaccine-induced Ebola virus glycoprotein-specific binding antibody concentrations at 21 days after the second dose (partial *r=*-0·204; appendix p 20). There was also no correlation observed between baseline Ebola glycoprotein-specific binding antibodies and the concentrations at 21 days after the second dose (partial *r*=-0·084; appendix p 21). None of the participants assessed for the presence of neutralising antibodies against the MVA vector backbone showed pre-existing MVA neutralising antibodies.

## Discussion

To our knowledge, this is the first clinical trial to report the safety and immunogenicity of a two-dose heterologous Ebola vaccine regimen (Ad26.ZEBOV and MVA-BN-Filo) in a paediatric population from a region affected by Ebola during the 2014–16 outbreak in west Africa. Consistent with previous studies in adults,^14,15,18^ this vaccine regimen was well tolerated in children and no safety concerns were identified. No deaths or serious adverse events attributed to the Ebola vaccines were observed, and there were no adverse events warranting discontinuation of study vaccinations. Vaccine-induced humoral immune responses were observed in the majority of study participants, assessed by both an Ebola virus glycoproteinspecific binding antibody assay and a pseudovirion neutralisation assay.

Overall, adverse events following vaccinations were mild and transient in all age cohorts. The proportion of study participants with at least one solicited local adverse event was higher in the Ebola vaccine group than in the control group for all age cohorts. Ad26.ZEBOV tended to be more reactogenic than MVA-BN-Filo, especially for solicited local adverse events, in the youngest cohort (1–3 years) and for solicited systemic adverse events in all age cohorts. Compared with adults, in whom headache, arthralgia, and myalgia were the predominant solicited systemic adverse events,^13^ the most frequent solicited systemic adverse events in participants aged 4–17 years were headache, fatigue, and chills. In participants aged 1–3 years, the most frequently reported solicited systemic adverse events were decreased appetite, decreased activity, and pyrexia. These events were mostly mild in severity (grade 1) and resolved within 24–48 h in most participants. The observed frequency of pyrexia in participants aged 1–3 years was higher than in the other two age cohorts, regardless of the vaccine given. This is consistent with previous findings in similar studies evaluating adenovirus-vectored and MVA-vectored vaccines in this age group.^19,20^ The common occurrence of pyrexia in children aged 1–3 years has also been reported following meningococcal and pneumococcal vaccinations in this age group, and is hypothesised to be caused by antigen-induced inflammatory responses.^21–25^

A change in haemoglobin concentration from baseline, assessed according to US FDA^16^ and the Division of Microbiology and Infectious Diseases toxicity grading systems,^26^ was observed in similar proportions of children aged 12–17 years in the Ebola vaccine and control groups. This observation illustrates the challenges commonly faced in adverse event reporting in paediatric vaccine trials done in low-income countries. When adopted as a gold standard method, international laboratory toxicity grading systems do not usually accommodate the epidemiological factors that can influence the physiological status of children in low-income countries. The fact that the so-called abnormal haemoglobin concentration values were within the acceptable normal ranges of a similar paediatric population in west Africa^17^ underscores the need for context-specific laboratory references for eligibility screening and adverse event reporting in paediatric vaccine trials in such settings. Nevertheless, given the high prevalence of malaria and other common childhood infectious diseases in the study area, the reduction in haemoglobin concentrations observed in our cohort might have been caused by these infections.^27^

There were no Ebola vaccine-related serious adverse events in this study. One participant in the 1–3 years age cohort developed severe thrombocytopenia following receipt of MenACWY as the first vaccine dose. Most episodes of vaccine-associated thrombocytopenia are asymptomatic, rare, and of limited duration; nevertheless, there are some reports of severe thrombocytopenia associated with bleeding following administration of the measles, mumps, and rubella vaccine and, sometimes, after administration of other routine childhood vaccines.^28^ Although some monovalent, non-replicating vaccines also have the potential to cause symptomatic thrombocytopenia,^29^ this event was not observed in any of the children who received the Ad26.ZEBOV and MVA-BN-Filo vaccine regimen.

Ebola virus glycoprotein-specific binding antibody responses were observed at 21 days after the second dose in at least 98% of the study participants in each age cohort, and these responses persisted for at least up to 1 year after the first dose in 96% of participants aged 1–3 years and around 70% of children aged 4–17 years. Relative to the older-age cohorts and adults in the study,^13^ the overall trend of higher immune responses in children aged 1–3 years is consistent with similar findings reported in a study of the chimpanzee adenovirus type-3 vectored Zaire Ebola glycoprotein vaccine (ChAd3-EBO-Z) in children,^20^ and in a study of an adenovirus-based malaria vaccine in The Gambia.^19,30^ Although a definite reason for this observation has yet to be established, possible suppression of immune responses by recurrent or multiple chronic infections, or both, such as malaria and helminth infections, which are prevalent in the west African setting and are known to affect humoral immune responses in older children,^31^ is a plausible explanation.

The correlation analysis between pre-existing Ad26 seropositivity and the Ebola virus glycoprotein-specific antibody responses following vaccination at an individual level indicates that pre-existing immunity against the Ad26 vector had no effect on vaccine-induced antibody responses. Therefore, pre-existing immunity against the Ad26 vector had no effect on the observed difference in Ebola virus glycoprotein-specific binding antibody concentrations for the children in the 1–3 years age cohort and the two older paediatric age cohorts (4–11 and 12–17 years). The two-dose Ebola vaccine regimen evaluated in this study induced robust Ebola virus glycoprotein-specific neutralising antibody responses. We observed a strong positive correlation between the Ebola virus glycoprotein-specific binding antibody concentrations and neutralising antibody titres at 21 days after the second dose, suggesting that most of the vaccine-induced binding antibodies also have a neutralising function.

A limitation of this study is that it focused only on safety and immunogenicity, despite the need to rapidly develop and roll out an efficacious, prophylactic Ebola vaccine to paediatric age groups. Since the study was done at a time when the west Africa Ebola outbreak had been brought under control, it was not possible to evaluate the efficacy of the vaccine regimen. Consequently, it was necessary to use statistical modelling (referred to as an immunobridging approach) to infer the potential clinical benefits induced by the Ad26.ZEBOV and MVA-BN-Filo regimen in study participants. This modelling involved correlating the magnitude of vaccine-elicited immune responses associated with protection in non-human primates with those observed in vaccinated human participants, including a pooled analysis specific to the paediatric population,^32,33^ following an approach similar to that used for establishing the efficacy of a vaccine against anthrax.^34^

In conclusion, this study shows that the Ad26.ZEBOV and MVA-BN-Filo Ebola vaccine regimen is safe and well-tolerated, induces strong and durable Ebola glycoprotein-specific binding antibody responses, and is likely to be protective against Ebola virus disease in adolescents and children. These findings contributed to the recent (July, 2020) approval by the EMA’s Committee for Medicinal Products for Human Use for use of the two-dose Ad26.ZEBOV and MVA-BN-Filo vaccine regimen in children,^12^ marking an important milestone in public health preparedness and the Ebola virus disease response for this susceptible age group. Given that Ebola virus disease affects a substantial proportion of children during outbreaks, the prophylactic use of this Ebola vaccine regimen could be beneficial in offering protection against Ebola virus disease and in mitigating the challenges of diagnostic dilemma, reduced chances of survival, and persistence of long-time sequelae in children.^3^

## Supplementary Material

Supplementary Material

## Figures and Tables

**Table 1:** Demographic and baseline characteristics of study participants by age cohort

	12–17 years age cohort			4–11 years age cohort			1–3 years age cohort		
	Ad26.ZEBOV and MVA-BN-FILO Ebola vaccine group (n=143*)	MenACWY and placebo control group (n=48)	Total (n=191)	Ad26.ZEBOV and MVA-BN-FILO Ebola vaccine group (n=144)	MenACWY and placebo control group (n=48)	Total (n=192)	Ad26.ZEBOV and MVA-BN-FILO Ebola vaccine group (n=144)	MenACWY and placebo control group (n=48)	Total (n=192)
Age at screening, years	14 (13–16)	14 (13–15)	14 (13–15)	8 (7–9)	8 (6–9)	8 (6–9)	2 (1–3)	2 (1–2)	2 (1–3)
Sex									
Male	74 (52%)	27 (56%)	101 (53%)	71 (49%)	22 (46%)	93 (48%)	77 (54%)	27 (56%)	104 (54%)
Female	69 (48%)	21 (44%)	90 (47%)	73 (51%)	26 (54%)	99 (52%)	67 (46%)	21 (44%)	88 (46%)
Height, cm	155 (148–161)	154 (146–160)	155 (147–161)	126 (118–135)	125 (119–133)	126 (118–133)	87 (80–92)	86 (79–92)	86 (80–92)
Weight, kg	45 (38–52)	43 (35–50)	45 (36–52)	24 (21–28)	25 (21–29)	24 (21–28)	12 (10–14)	12 (10–14)	12 (10–14)
Body-mass index, kg/m^2^†	18 (17–20)	18 (16–19)	18 (17–20)	NA	NA	NA	NA	NA	NA

Data are median (IQR) or n (%).

* One participant in this group received Ad26.ZEBOV followed by placebo and was excluded from further analysis.

† BMI is not a reliable reflection of growth index in children and was therefore not calculated for children aged younger than 12 years. Ad26.ZEBOV=adenovirus type 26 vector-based vaccine encoding the Ebola virus glycoprotein. MenACWY=meningococcal quadrivalent (serogroups A, C, W135, and Y) conjugate vaccine. MVA-BN-Filo=modified vaccinia Ankara vector-based vaccine, encoding glycoproteins from the Ebola virus, Sudan virus, and Marburg virus, and the nucleoprotein from the Tai Forest virus. NA=not assessed.

## Data Availability

Janssen has an agreement with the Yale Open Data Access (YODA) Project to serve as the independent review panel for evaluation of requests for clinical study reports and participant-level data from investigators and physicians for scientific research that will advance medical knowledge and public health. Data will be made available following publication and approval by YODA of any formal requests with a defined analysis plan. For more information on this process, or to make a request, please visit The YODA Project website at http://yoda.yale.edu. The data sharing policy of Janssen Pharmaceutical Companies of Johnson & Johnson is available at https://www.janssen.com/clinical-trials/transparency. The clinical study protocol for this study is available in the appendix (pp 26–155). We have also clearly reported the participant data in the text, tables, figures, and appendices of this manuscript. Individual participant data, including data dictionaries, will not be shared.

## Research in context

### Evidence before this study

Ebola virus disease is a highly contagious infection. Children accounted for approximately 20% of infected people during the 2014–16 Ebola outbreak in west Africa and approximately 30% of infected people in the 2018–20 outbreak in the Democratic Republic of the Congo. No Ebola vaccine studies in children were done before the 2014–16 outbreak. We searched MEDLINE and Embase from database inception to April 3, 2020, using the search string “Ebola” AND “(vaccin* OR immunis* OR immuniz*)” AND “(trial* OR study)” AND “(child* OR infant* OR pediatr* OR paediatr* OR adolescen*)”. We searched for peer-reviewed articles reporting Ebola vaccine trials in children and adolescents, with no language restrictions. Public clinical trial registries and a WHO report summarising candidate Ebola vaccines being studied as of August 19, 2019, were also searched.

The database searches yielded 190 citations. After screening the titles or abstracts and removing duplicates, we identified four relevant publications. One was a randomised, open-label, phase 1 trial done in Gabon, which evaluated a recombinant vesicular stomatitis virus-vectored vaccine expressing an Ebola virus surface glycoprotein (rVSV-ZEBOV-GP [ERVEBO]) in 20 children aged 6–12 years and 20 adolescents aged 13–17 years. The vaccine had an acceptable safety and immunogenicity profile, but children and adolescents in this study had a higher amount of vaccine replication than adults, which led to shedding of the vaccine in the saliva and urine. To address this concern, the authors recommended a low dose of the vaccine for children and adolescents. Given the promising safety findings obtained from this trial, two subsequent studies evaluated a ring vaccination approach in 303 children and adolescents in Guinea in 2015 and 2016, respectively. Adverse event data indicated no safety concerns in children and adolescents. The vaccine used in this study (rVSV-ZEBOV-GP) has been granted conditional authorisation by the European Medicines Agency (EMA) and was licenced by the US Food and Drug Administration for use in adults who are at risk of Ebola virus disease. The final study identified was a randomised, observer-blind, placebo-controlled, phase 2 trial evaluating the safety, reactogenicity, and immunogenicity of a chimpanzee adenovirus type 3-vectored Zaire Ebola glycoprotein vaccine (ChAd3-EBO-Z) in children in Mali and Senegal. A total of 600 children (equally divided among 1–5 years, 6–12 years, and 13–17 years age cohorts) were randomly assigned (1:1) to receive either ChAd3-EBO-Z at day 0 and meningococcal serogroups A, C, W135, Y tetanus toxoid conjugate vaccine (MenACWY-TT) at 6 months, or MenACWY-TT at day 0 and ChAd3-EBO-Z at 6 months. The ChAd3-EBO-Z vaccine was tolerable and immunogenic. However, increased reactogenicity and stronger immune responses to this vaccine were observed in the youngest age cohort (1–5 years). The authors reported that this result could be explained by the youngest age cohort having received a higher dose of the vaccine relative to their body mass than the other two cohorts.

### Added value of this study

To our knowledge, this is the first study evaluating a two-dose heterologous Ebola virus vaccine regimen, comprising the adenovirus type 26 vector-based vaccine encoding the Ebola virus glycoprotein (Ad26.ZEBOV) and the modified vaccinia Ankara vector-based vaccine, encoding glycoproteins from the Ebola virus, Sudan virus, and Marburg virus, and the nucleoprotein from the Tai Forest virus (MVA-BN-Filo), in a randomised, double-blind, controlled trial in a paediatric population. Immune responses can be enhanced if the same antigen is delivered successively through 2 different vectors. This heterologous approach is a potent way of inducing both humoral and cellular immune responses and generally elicits higher immune responses than repeated administration of the same vaccine (homologous approach). We found that the two-dose Ebola vaccine regimen was well tolerated by the study participants, and identified no safety concerns. This vaccine regimen also induced strong humoral immune responses that persisted for at least 12 months after the first vaccination.

### Implications of all the available evidence

To date, three candidate vaccine regimens against Ebola virus disease (rVSV-ZEBOV-GP, ChAd3-EBO-Z, and Ad26.ZEBOV and MVA-BN-Filo) have been evaluated in children and adolescents, all of which were found to have acceptable safety and immunogenicity profiles. Before July, 2020, no licenced vaccine was available against Ebola virus disease for this susceptible age group. Data from our study and the trial of the same regimen in adults contributed to the recent (July, 2020) approval and marketing authorisation of the two-dose Ad26.ZEBOV and MVA-BN-Filo vaccine regimen by the EMA’s Committee for Medicinal Products for Human Use for use in children and adults. Our report highlights the important progress that has been made in the development of an Ebola virus disease vaccine regimen for use in children, and contributes to public health preparedness and response.

## References

[1] Centres for Disease Control and Prevention (2019). 2014–2016 Ebola outbreak in west Africa.

[2] Lo TQ, Marston BJ, Dahl BA, De Cock KM (2017). Ebola: anatomy of an epidemic. Annu Rev Med.

[3] Agua-Agum J, Ariyarajah A, Blake IM (2015). Ebola virus disease among children in west Africa. N Engl J Med.

[4] Centres for Disease Control and Prevention (2019). Cost of the Ebola epidemic.

[5] WHO (2020). Ebola virus disease Democratic Republic of the Congo: external siguation report 97/2020.

[6] Matz KM, Marzi A, Feldmann H (2019). Ebola vaccine trials: progress in vaccine safety and immunogenicity. Expert Rev Vaccines.

[7] Henao-Restrepo AM, Camacho A, Longini IM (2017). Efficacy and effectiveness of an rVSV-vectored vaccine in preventing Ebola virus disease: final results from the Guinea ring vaccination, open-label, cluster-randomised trial (Ebola Ca Suffit!). Lancet.

[8] Shears P, Garavan C (2020). The 2018/19 Ebola epidemic the Democratic Republic of the Congo (DRC): epidemiology, outbreak control, and conflict. Infect Prev Pract.

[9] European Commission (2019). Vaccine against Ebola: commission grants first-ever market authorisation.

[10] US Food and Drug Administration (2019). First FDA-approved vaccine for the prevention of Ebola virus disease, marking a critical milestone in public health preparedness and response.

[11] WHO (2019). Strategic Advisory Group of Experts (SAGE) on Immunization interim recommendations on vaccination against Ebola virus disease (EVD).

[12] European Commission (2020). Vaccine against Ebola: commission grants new market authorisations.

[13] Ishola D, Manno D, Afolabi MO (2021). Safety and long-term immunogenicity of the two-dose heterologous Ad26.ZEBOV and MVA-BN-Filo Ebola vaccine regimen in adults in Sierra Leone: a combined open-label, non-randomised stage 1 trial, and a randomised, double-blind, controlled stage 2 trial. Lancet Infect Dis.

[14] Mutua G, Anzala O, Luhn K (2019). Safety and immunogenicity of a 2-dose heterologous vaccine regimen with Ad26.ZEBOV and MVA-BN-Filo Ebola vaccines: 12-month data from a phase 1 randomized clinical trial in Nairobi, Kenya. J Infect Dis.

[15] Anywaine Z, Whitworth H, Kaleebu P (2019). Safety and immunogenicity of a 2-dose heterologous vaccination regimen with Ad26.ZEBOV and MVA-BN-Filo Ebola vaccines: 12-month data from a phase 1 randomized clinical trial in Uganda and Tanzania. J Infect Dis.

[16] US Food and Drug Administration (2007). Guidance for industry: toxicity grading scale for healthy adult and adolescent volunteers enrolled in preventive vaccine clinical trials.

[17] Dosoo DK, Asante KP, Kayan K (2014). Biochemical and hematologic parameters for children in the middle belt of Ghana. Am J Trop Med Hyg.

[18] Pollard AJ, Launay O, Lelievre JD (2020). Safety and immunogenicity of a two-dose heterologous Ad26.ZEBOV and MVA-BN-Filo Ebola vaccine regimen in adults in Europe (EBOVAC2): a randomised, observer-blind, participant-blind, placebo-controlled, phase 2 trial. Lancet Infect Dis.

[19] Afolabi MO, Tiono AB, Adetifa UJ (2016). Safety and immunogenicity of ChAd63 and MVA ME-TRAP in west African children and infants. Mol Ther.

[20] Tapia MD, Sow SO, Mbaye KD (2020). Safety, reactogenicity, and immunogenicity of a chimpanzee adenovirus vectored Ebola vaccine in children in Africa: a randomised, observer-blind, placebo-controlled, phase 2 trial. Lancet Infect Dis.

[21] US Food and Drug Administration (2020). BLA clinical review memorandum.

[22] GlaxoSmithKline (2020). Highlights of prescribing information: Menveo.

[23] Mendoza YG, Garric E, Leach A (2019). Safety profile of the RTS,S/ AS01 malaria vaccine in infants and children: additional data from a phase III randomized controlled trial in sub-Saharan Africa. Human Vacc Immunother.

[24] Odusanya OO, Kuyinu YA, Kehinde OA (2014). Safety and immunogenicity of 10-valent pneumococcal non typeable *Haemophilus influenzae* protein D conjugate vaccine (PHiD-CV) in Nigerian children. Hum Vaccin Immunother.

[25] Thompson A, Gurtman A, Patterson S (2013). Safety of 13-valent pneumococcal conjugate vaccine in infants and children: metaanalysis of 13 clinical trials in 9 countries. Vaccine.

[26] National Institute of Allergy and Infectious Diseases (2007). Division of Microbiology and Infectious Diseases (DMID) pediatric toxicity tables November 2007 draft.

[27] Wirth JP, Rohner F, Woodruff BA (2016). Anemia, micronutrient deficiencies, and malaria in children and women in Sierra Leone prior to the Ebola outbreak—findings of a cross-sectional study. PLoS One.

[28] Mantadakis E, Farmaki E, Buchanan GR (2010). Thrombocytopenic purpura after measles-mumps-rubella vaccination: a systematic review of the literature and guidance for management. J Pediatr.

[29] Cecinati V, Principi N, Brescia L, Giordano P, Esposito S (2013). Vaccine administration and the development of immune thrombocytopenic purpura in children. Hum Vaccin Immunother.

[30] Bliss CM, Drammeh A, Bowyer G (2017). Viral vector malaria vaccines induce high-level T cell and antibody responses in west African children and infants. Mol Ther.

[31] Mwangi TW, Bethony JM, Brooker S (2006). Malaria and helminth interactions in humans: an epidemiological viewpoint. Ann Trop Med Parasitol.

[32] Bockstal V, Roozendaal R, Effelterre TV (2018). Immunobridging approach to assess clinical benefit as the basis for licensure of the monovalent Ebola vaccine.

[33] Roozendaal R, Hendriks J, van Effelterre T (2020). Nonhuman primate to human immunobridging to infer the protective effect of an Ebola virus vaccine candidate. NPJ Vaccines.

[34] Schiffer JM, Chen L, Dalton S, Niemuth NA, Sabourin CL, Quinn CP (2015). Bridging non-human primate correlates of protection to reassess the anthrax vaccine adsorbed booster schedule in humans. Vaccine.

