# Supplementary material for: Safety and immunogenicity of the two-dose heterologous Ad26.ZEBOV and MVA-BN-Filo Ebola vaccine regimen in children in Sierra Leone: a randomised, double-blind, controlled trial
Source: Lancet Infect Dis. Author manuscript; Available in PMC 2022 Sep 7. (PMC7613317; doi:10.1016/S1473-3099(21)00128-6)
Supplement: Supplementary Material [file EMS152542-supplement-Supplementary_Material.pdf]

THE LANCET
Infectious Diseases

Supplementary appendix

This appendix formed part of the original submission and has been peer reviewed. We post it as supplied by the authors.

Supplement to: Afolabi MO, Ishola D, Manno D, et al. Safety and immunogenicity of the two-dose heterologous Ad26.ZEBOV and MVA-BN-Filo Ebola vaccine regimen in children in Sierra Leone: a randomised, double-blind, controlled trial. *Lancet Infect Dis* 2021; published online Sept 13. https://doi.org/10.1016/S1473-3099(21)00128-6.

Safety and immunogenicity of a two-dose Ad26. ZEBOV, MVA-BN-Filo Ebola vaccine in children in Sierra Leone: a randomised, double-blind, controlled trial

Supplementary materials

Table of Contents

1. SUPPLEMENTARY METHODS 2

1.1. Assessment of safety and reactogenicity 2

1.2. Assessment of immunogenicity 3

1.2.1. Determination of neutralising antibody activity in an EBOV GP pseudovirion neutralisation assay 3

2. SUPPLEMENTARY RESULTS 5

2.1. ***Table S1: Summary of solicited adverse events in study participants*** *........ 5*

2.2. ***Table S2: Solicited local adverse events in study participants*** *........ 6*

2.3. ***Table S3: Solicited systemic adverse events in study participants*** *........ 7*

2.4. ***Table S4: Unsolicited adverse events (AEs) in study participants reported between dose 1 vaccination and 28 days post-dose 1, and between dose 2 vaccination and 28 days post-dose 2*** *........ 9*

2.5. ***Table S5: Serious adverse events by system organ class and dictionary-derived term in adolescents and children*** *10*

2.7. ***Table S7: Geometric mean (with 95% CI) EBOV GP-specific binding antibody concentrations before and after vaccination and the responder rate in study participants*** *........ 14*

2.8. ***Table S8: Geometric mean (with 95% CI) EBOV GP-specific neutralizing antibody concentrations before and after vaccination and the responder rate in study participants*** *........ 16*

2.8. ***Table S9: Ad26 neutralising antibodies (Ad26 VNA, IC_{90} titre): Geometric mean titre and sample interpretation in study participants per protocol analysis set*** *........ 18*

2.9. ***Figure S1: Correlation between EBOV GP-specific binding antibody concentrations and EBOV GP-specific neutralising antibody titres in study participants*** *........ 19*

2.10. ***Figure S2: Correlation between pre-vaccination Ad26 neutralising antibody titres and EBOV GP-specific binding antibody concentrations in study participants*** *........ 20*

2.11. ***Figure S3: Correlation between EBOV GP-specific binding antibody concentrations at baseline and post-vaccination in study participants*** *........ 21*

3. EBL3001 STUDY GROUP 22

4. STUDY PROTOCOL 26

1. SUPPLEMENTARY METHODS

1.1. Assessment of safety and reactogenicity

After each vaccination, study participants were directly observed in the trial clinics for 30 minutes and then followed up at home. Trained field assistants visited the study participants at home daily for seven days after each vaccination to provide a standardised, purpose-designed reactogenicity diary card to the study child and/or their parent/guardian. For the 12–17- and 4–11-year old cohort, the reactogenicity symptoms to be recorded included a history of fever, headache, myalgia, arthralgia, vomiting, diarrhoea, or loss of appetite, while for 1–3-year-old children, the symptoms to be recorded included fever, excessive crying, irritability, reduced oral intake, and reduced activities. The field assistants also examined children for expected local adverse events (AEs) (swelling, tenderness, limitation of arm movement, redness). Pain at the injection site was graded on a scale of 0 to 3 (where 0 = no pain, 1 = painful to touch, 2 = pain when the arm is touched, and 3 = severe pain at rest).

Safety assessments were performed for all study participants on day 8 after dose 1 vaccination, on day 57 (before dose 2 vaccination was administered) and on day 64 (day 8 after dose 2 vaccination). Clinical evaluations at these clinic visits included information on solicited and unsolicited symptoms, measurement of vital signs and assessment of the injection site. Blood samples were collected at these visits to determine complete blood count, alanine aminotransferase, aspartate aminotransferase and serum creatinine. AEs were graded by intensity and judged for relatedness to the study vaccine as indicated in supplement table S4.

Grade 1 (mild) AEs were tolerated easily, causing minimal or no interference with usual social and functional activities. Grade 2 (moderate) AEs were those causing greater than minimal interference with usual social and functional activities. Grade 3 (severe) AEs prevented usual social and functional activities.

Swelling, redness, and fever had specific definitions not based on interference with daily activities. Injection site swelling and redness were graded based on their widest dimension: mild, 0–20 mm; moderate, 20–50 mm; and severe, >50 mm. Fever was classified as severe if the axillary temperature was ≥38.9°C. For laboratory tests, toxicity grading was adapted to normal reference ranges determined for a paediatric population in the West Africa sub-region.[1]

To further safeguard participant safety, the following pausing rules for the 12–17-year-old cohort were devised: (i) death of any participant considered to be at least possibly related to the study vaccine; OR (ii) an anaphylactic reaction within 24 hours of vaccination or the presence of generalized urticaria within 72 hours of vaccination considered to be at least possibly related to the study vaccine; OR (iii) a life-threatening or other serious AE in any participant considered to be at least possibly related to the study vaccine. For 1–3 and 4–11 year cohorts, additional pausing rules included (iv) three or more participants experiencing a severe (grade 3) (non-serious) unsolicited AE (of the same type) considered to be related to a study vaccine that persisted for 3 or more days; (v) three or more participants experiencing a persistent (upon repeat testing) severe (grade 3) (non-serious) laboratory abnormality (including unexplained haematuria) related to the same laboratory parameter and considered to be related to a study vaccine; OR (vi) three or more participants experiencing the same severe (grade 3) (non-serious) solicited systemic reaction considered to be related to a study vaccine that persisted for 3 or more days.

The toxicity scales used for the assessments of clinical laboratory values were based on the United States FDA Toxicity Grading Scale for Healthy Adults and Adolescent Volunteers Enrolled in Preventive Vaccine Clinical Trials[2] and the Division of Microbiology and Infectious Diseases (DMID) Toxicity Tables for use in trials enrolling children greater than 3 months of age.[3] In participants aged ≥12 years, any change in haemoglobin from baseline was considered a laboratory abnormality, even if the haemoglobin value was within laboratory normal ranges and graded based on the FDA toxicity grading scale. In participants aged <12 years, haemoglobin values were assessed on the absolute value and graded based on the DMID Toxicity Tables.

1.2. Assessment of immunogenicity

Immunoglobulin G responses against EBOV GP were analysed using the EBOV GP (Kikwit) Filovirus Animal Non-Clinical Group (FANG) ELISA at Q2 Solutions – Vaccine Testing Laboratory, San Juan Capistrano, CA. In a randomly selected subset of the study participants, the neutralising activity of vaccine-induced antibody responses was assessed using a EBOV GP (Makona) pseudovirion neutralisation assay (psVNA) at Monogram Biosciences, San Francisco, CA.

Neutralising antibodies against the Ad26 vector backbone were measured at baseline using an Ad26-specific virus neutralization assay (Ad26 VNA) by inhibition of Ad26 infection of A549 cells. The assay was validated as reported in validation report NL-SYR-1003-10, 'Validation of the Ad26 neutralisation assay for human serum and was performed by the Sponsor based on the Standard Operating Procedure (SOP): 'Determination of Neutralizing Antibodies Against Adenovirus Type 5, 26 and 36 in Human and Rabbit Serum Samples'.

Antibodies against the MVA vector backbone were measured using a plaque reduction neutralisation test (PRNT). The assay was validated in accordance with International Council for Harmonisation (ICH) Q2 (R1) (Method Validation Report: Human Plaque Reduction Neutralization Assay Using Vaccinia Virus Western Reserve, Document No. 8200478). The assay was performed at Bavarian Nordic, Martinsried, Germany based on the Bavarian Nordic SOP: Human Plaque Reduction Neutralisation Test Using Vaccinia Western Reserve.

1.2.1. Determination of neutralising antibody activity in an EBOV GP pseudovirion neutralisation assay

In order to assess the functionality of vaccine-induced antibody responses, a pseudovirion neutralisation assay (psVNA was developed at Monogram Biosciences (San Francisco, CA, USA) and was validated with human serum. Samples for the current report were assayed at Monogram Biosciences according to the SOP Crucell EBOV Neutralization Assay.

Pseudovirions expressing the glycoprotein of an EBOV isolate from the 2014 outbreak (Makona variant) were produced in human embryonic kidney 293 (HEK293) cells by transfection with a GP expression plasmid and a vector encoding a firefly luciferase gene and all of the human immunodeficiency virus (HIV) type 1 genes required for viral replication, except for the envelope gene. Sera were pre-treated to remove any nonspecific neutralising factors. A fixed amount of pseudovirions was mixed with a series of serial dilutions of serum samples. Following incubation, the samples were transferred to a HEK293 cell monolayer. The inhibition of pseudovirion infection was measured by luciferase reporter gene expression. The assay responses of the serially diluted samples were plotted in a 4-parameter logistic regression curve and the 50% inhibitory concentration (IC_{50}) of each curve was reported as a neutralisation titre for each serum sample.

A psVNA result (IC_{50} titre) was considered positive if the specific IC_{50} titre was more than three times amphotropic murine leukaemia virus (aMLV) and above the assay-specific lower limit of quantitation (LLOQ). Values that were less than three

times aMLV or below the LLOQ were imputed with LLOQ/2 (120/2). For the calculation of fold increases, values that were less than three times aMLV or below the LLOQ were imputed with the LLOQ. The psVNA values were $\log_{10}$-transformed before further handling. The $\log_{10}$-transformed values were used throughout the entire analysis.

References:

1. Dosoo DK, Asante KP, Kayan K, et al. Biochemical and hematologic parameters for children in the middle belt of Ghana. Am J Trop Med Hyg 2014; **90**: 767–73.
2. US FDA. Guidance for Industry: Toxicity Grading Scale for Healthy Adult and Adolescent Volunteers Enrolled in Preventive Vaccine Clinical Trials. Revised September 2007. Available at: https://www.fda.gov/regulatory-information/search-fda-guidance-documents/toxicity-grading-scale-healthy-adult-and-adolescent-volunteers-enrolled-preventive-vaccine-clinical. Accessed 23 June 2020.
3. Division of Microbiology and Infectious Diseases (DMID) Pediatric Toxicity Tables November 2007 draft. Available at: https://www.niaid.nih.gov/sites/default/files/dmidpedtox.pdf. Accessed 23 June 2020

2. SUPPLEMENTARY RESULTS

2.1. *Table S1*: Summary of solicited adverse events in study participants

**Participants with at least one solicited event**	**12–17 Years**		**4–11 Years**		**1–3 Years**	
**Post-dose 1**	**Ad26.ZEBOV**	**MenACWY**	**Ad26.ZEBOV**	**MenACWY**	**Ad26.ZEBOV**	**MenACWY**
n	143	48	144	48	144	48
Solicited AE, n (%)	55 (38)	16 (33)	60 (42)	16 (33)	49 (34)	16 (33)
Solicited AE with severity grade 3	2 (1)	1 (2)	0	0	1 (1)	0
Solicited local AE	14 (10)	3 (6)	30 (21)	2 (4)	21 (15)	5 (10)
Solicited local AE with severity grade 3	0	0	0	0	0	0
Solicited systemic AE	52 (36)	14 (29)	45 (31)	15 (31)	36 (25)	12 (25)
Solicited systemic AE with severity grade 3	2 (1)	1 (2)	0	0	1 (1)	0
**Post-dose 2**	**MVA-BN-Filo**	**Placebo**	**MVA-BN-Filo**	**Placebo**	**MVA-BN-Filo**	**Placebo**
n	142	46	143	48	143	48
Solicited AE, n (%)	38 (27)	6 (13)	45 (31)	11 (23)	29 (20)	14 (29)
Solicited AE with severity grade 3	0	0	0	0	1 (1)	2 (4)
Solicited local AE	21 (15)	1 (2)	22 (15)	5 (10)	7 (5)	0
Solicited local AE with severity grade 3	0	0	0	0	0	0
Solicited systemic AE	26 (18)	6 (13)	27 (19)	8 (17)	23 (16)	14 (29)
Solicited systemic AE with severity grade 3	0	0	0	0	1 (1)	2 (4)

Vaccines: Ad26.ZEBOV at a dose of 5×10^{10} vp; MVA-BN-Filo at a dose of 1×10^{8} Inf.U. Control: Meningococcal quadrivalent (serogroups A, C, W135 and Y) conjugate vaccine (MenACWY; dose 1), Placebo (dose 2). AE=adverse event; n=number of participants with data; n (%)=number (percentage) of participants with 1 or more events, where the denominator is the number of participants with available reactogenicity data after the given dose.

2.2. *Table S2*: Solicited local adverse events in study participants

Participants with at least one event		12–17 Years		4–11 Years		1–-3 Years	
**Post-dose 1**		**Ad26.ZEBOV**	**MenACWY**	**Ad26.ZEBOV**	**MenACWY**	**Ad26.ZEBOV**	**MenACWY**
**n**		143	48	144	48	144	48
Any solicited local event, n (%)	Any	14 (10)	3 (6)	30 (21)	2 (4)	21 (15)	5 (10)
Injection-site erythema	Any	0	0	0	0	0	0
Injection-site pain	Any	13 (9)	3 (6)	30 (21)	2 (4)	20 (14)	5 (10)
Injection-site pruritus	Any	1 (1)	0	0	0	3 (2)	0
Injection-site swelling	Any	0	0	0	0	0	0
**Post-dose 2**		**MVA-BN-Filo**	**Placebo**	**MVA-BN-Filo**	**Placebo**	**MVA-BN-Filo**	**Placebo**
**n**		142	46	143	48	143	48
Any solicited local event, n (%)	Any	21 (15)	1 (2)	22 (15)	5 (10)	7 (5)	0
Injection-site erythema	Any	0	0	0	0	0	0
Injection-site pain	Any	21 (15)	1 (2)	20 (14)	5 (10)	7 (5)	0
Injection-site pruritus	Any	0	0	1 (1)	0	0	0
Injection-site swelling	Any	0	0	1 (1)	0	0	0

Most events were grade 1 or 2; only the total number of events and grade 3 are displayed.

Vaccines: Ad26.ZEBOV at a dose of $5x10^{10}$ vp; MVA-BN-Filo at a dose of $1x10^{8}$ Inf.U. Control: Meningococcal quadrivalent (serogroups A, C, W135 and Y) conjugate vaccine (MenACWY; dose 1), Placebo (dose 2).

n=number of participants with data; n (%)=number (percentage) of participants with 1 or more events, where the denominator is the number of participants with available reactogenicity data after the given dose.

=

2.3. *Table S3*: Solicited systemic adverse events in study participants

**Participants with at least one event**		**12–17 Years**		**4–11 Years**		**1–3 Years**	
**Post-dose 1**		**Ad26.ZEBOV**	**MenACWY**	**Ad26.ZEBOV**	**MenACWY**	**Ad26.ZEBOV**	**MenACWY**
**n**		143	48	144	48	144	48
Any solicited systemic event, n (%)	Any	52 (36)	14 (29)	45 (31)	15 (31)	36 (25)	12 (25)
	Grade 3	2 (1)	1 (2)	0	0	1 (1)	0
Arthralgia	Any	5 (3)	0	7 (5)	1 (2)	NA	NA
Chills	Any	15 (10)	1 (2)	17 (12)	5 (10)	NA	NA
Fatigue	Any	20 (14)	1 (2)	15 (10)	8 (17)	NA	NA
Headache	Any	41 (29)	11 (23)	34 (24)	14 (29)	NA	NA
Myalgia	Any	12 (8)	1 (2)	6 (4)	1 (2)	NA	NA
Nausea	Any	1 (1)	1 (2)	8 (6)	2 (4)	NA	NA
Pyrexia	Any	4 (3)	1 (2)	6 (4)	2 (4)	16 (11)	4 (8)
	Grade 3	2 (1)	1 (2)	0	0	1 (1)	0
Decreased activity	Any	NA	NA	NA	NA	19 (13)	6 (13)
Decreased appetite	Any	NA	NA	NA	NA	20 (14)	3 (6)
Irritability	Any	NA	NA	NA	NA	15 (10)	4 (8)
Vomiting	Any	NA	NA	NA	NA	9 (6)	1 (2)
**Post-dose 2**		**MVA-BN-Filo**	**Placebo**	**MVA-BN-Filo**	**Placebo**	**MVA-BN-Filo**	**Placebo**
**n**		142	46	143	48	143	48
Any solicited systemic event, n (%)	Any	26 (18)	6 (13)	27 (19)	8 (17)	23 (16)	14 (29)
	Grade 3	0	0	0	0	1 (1)	2 (4)
Arthralgia	Any	5 (4)	2 (4)	4 (3)	0	NA	NA
Chills	Any	9 (6)	0	4 (3)	0	NA	NA
Fatigue	Any	8 (6)	1 (2)	9 (6)	0	NA	NA
Headache	Any	15 (11)	3 (7)	21 (15)	8 (17)	NA	NA

Participants with at least one event		12–17 Years		4–11 Years		1–3 Years	
Myalgia	Any	3 (2)	1 (2)	6 (4)	1 (2)	NA	NA
Nausea	Any	0	0	5 (3)	1 (2)	NA	NA
Pyrexia	Any	0	0	5 (3)	0	12 (8)	7 (15)
	Grade 3	0	0	0	0	1 (1)	0
Decreased activity	Any	NA	NA	NA	NA	12 (8)	5 (10)
	Grade 3	NA	NA	NA	NA	0	1 (2)
Decreased appetite	Any	NA	NA	NA	NA	14 (10)	6 (13)
	Grade 3	NA	NA	NA	NA	0	1 (2)
Irritability	Any	NA	NA	NA	NA	6 (4)	3 (6)
Vomiting	Any	NA	NA	NA	NA	8 (6)	4 (8)
	Grade 3	NA	NA	NA	NA	0	1 (2)

Vaccines: Ad26.ZEBOV at a dose of $5x10^{10}$ vp; MVA-BN-Filo at a dose of $1x10^{8}$ Inf.U. Control: Meningococcal quadrivalent (serogroups A, C, W135 and Y) conjugate vaccine (MenACWY; dose 1), Placebo (dose 2).

AE=adverse event; n=number of participants with data; n (%)=number (percentage) of participants with 1 or more events, where the denominator is the number of participants with available reactogenicity data after the given dose; NA=not applicable.

The majority of events were grade 1 or 2; only the total number of events and grade 3 are displayed.

2.4. ***Table S4*: Unsolicited adverse events (AEs) in study participants reported between dose 1 vaccination and 28 days post-dose 1, and between dose 2 vaccination and 28 days post-dose 2**

**Participants with at least one event**	**12–17 Years**		**4–11 Years**		**1–3 Years**	
**Post-dose 1**	**Ad26.ZEBOV**	**MenACWY**	**Ad26.ZEBOV**	**MenACWY**	**Ad26.ZEBOV**	**MenACWY**
**n**	143	48	144	48	144	48
Unsolicited AE	54 (38)	20 (42)	60 (42)	18 (38)	88 (61)	28 (58)
Infections and infestations	36 (25)	12 (25)	53 (37)	15 (31)	78 (54)	25 (52)
Malaria	21 (15)	7 (15)	39 (27)	11 (23)	53 (37)	14 (29)
Unsolicited AE with severity grade 3 as worst grade	5 (3)	4 (8)	0	0	5 (3)	0
**Post-dose 2**	**MVA-BN-Filo**	**Placebo**	**MVA-BN-Filo**	**Placebo**	**MVA-BN-Filo**	**Placebo**
**n**	142	46	143	48	143	48
Unsolicited AE	49 (35)	13 (28)	46 (32)	13 (27)	92 (64)	31 (65)
Infections and infestations	31 (22)	6 (13)	31 (22)	8 (17)	85 (59)	28 (58)
Malaria	19 (13)	4 (9)	16 (11)	5 (10)	59 (41)	19 (40)
Unsolicited AE with severity grade 3 as worst grade	4 (3)	3 (7)	0	0	10 (7)	2 (4)

This table only includes AEs that were reported between dose 1 vaccination and 28-days post-dose 1, and between dose 2 vaccination and 28-days post-dose 2.

Vaccines: Ad26.ZEBOV at a dose of $5x10^{10}$ vp; MVA-BN-Filo at a dose of $1x10^{8}$ Inf.U. Control: Meningococcal quadrivalent (serogroups A, C, W135 and Y) conjugate vaccine (MenACWY; dose 1), Placebo (dose 2). AE=adverse event

2.5. *Table S5*: **Serious adverse events by system organ class and dictionary-derived term in adolescents and children**

	Stage 2: 12–17 Years		Stage 2: 4–11 Years		Stage 2: 1–3 Years	
MedDRA System Organ Class<br>Dictionary-derived Term	**Ad26.ZEBOV, MVA-BN-Filo**	**Control**	**Ad26.ZEBOV, MVA-BN-Filo**	**Control**	**Ad26.ZEBOV, MVA-BN-Filo**	**Control**
**Entire study**	143	48	144	48	144	48
Any event, n (%)	0	1 (2)	5 (3)	0	15 (10)	3 (6)
Infections and infestations	0	1 (2)	4 (3)	0	15 (10)	2 (4)
Malaria	0	0	2 (1)	0	14 (10)	2 (4)
Sepsis	0	0	0	0	6 (4)	0
Pneumonia	0	0	0	0	4 (3)	0
Meningitis bacterial	0	0	0	0	1 (1)	1 (2)
Bronchiolitis	0	0	0	0	1 (1)	0
Gastroenteritis	0	0	1 (1)	0	0	0
Osteomyelitis chronic	0	0	1 (1)	0	0	0
Peritonitis	0	0	1 (1)	0	0	0
Postoperative wound infection	0	0	1 (1)	0	0	0
Respiratory tract infection	0	0	1 (1)	0	0	0
Subcutaneous abscess	0	0	0	0	1 (1)	0
Typhoid fever	0	1 (2)	0	0	0	0
Blood and lymphatic system disorders	0	0	1 (1)	0	5 (3)	1 (2)
Anaemia	0	0	1 (1)	0	4 (3)	0
Iron deficiency anaemia	0	0	0	0	1 (1)	0
Thrombocytopenia	0	0	0	0	0	1 (2)
Nervous system disorders	0	0	0	0	1 (1)	0
Febrile convulsion	0	0	0	0	1 (1)	0
Respiratory, thoracic and mediastinal disorders	0	0	1 (1)	0	0	0
Asthma	0	0	1 (1)	0	0	0

Children aged less than 2 years at the time of the initial vaccination (randomization) were planned to receive a third vaccination on the 3-month post-dose 2 visit with either MenACWY (control arm) or placebo (Ad26, MVA arm). Only serious AEs and immediate reportable events were collected after the third vaccination. Unsolicited AEs reported after the third vaccination are assigned to the post-dose 2 follow up period for statistical analysis.
Vaccines: Ad26.ZEBOV at a dose of $5x10^{10}$ vp; MVA-BN-Filo at a dose of $1x10^{8}$ Inf.U. Control: Meningococcal quadrivalent (serogroups A, C, W135 and Y) conjugate vaccine (MenACWY; dose 1), Placebo (dose 2).
n=number of participants with data; n (%)=number (percentage) of participants with 1 or more events, where the denominator is the number of participants with available reactogenicity data after the given dose.

2.6. Table ***S6*: Laboratory adverse events in study participants between dose 1 vaccination and 28-days post-dose 1, and between dose 2 vaccination and 28-days-post-dose 2**

		12–17 Years		4–11 Years		1–3 Years	
**Post-dose 1**		**Ad26.ZEBOV**	**MenACWY**	**Ad26.ZEBOV**	**MenACWY**	**Ad26.ZEBOV**	**MenACWY**
**n**		143	48	144	48	144	48
Chemistry, n (%)							
Alanine Aminotransferase	Any	0[a]	0	1 (1)[b]	2 (4)	2 (1)	1 (2)
Aspartate Aminotransferase	Any	0[a]	1 (2)	0[b]	3 (6)	2 (1)	0
Haematology							
Haemoglobin	Grade 2	NA	NA	2 (1)	1 (2)	1 (1)	0
	Grade 3	NA	NA	0	0	1 (1)	0
Haemoglobin change from baseline	Grade 1	87 (61)[a]	31 (65)	NA	NA	NA	NA
	Grade 2	6 (4)[a]	2 (4)	NA	NA	NA	NA
	Grade 3	2 (1)[a]	2 (4)	NA	NA	NA	NA
Platelets	Grade 2	0[a]	0	0	0	1 (1)	1 (2)
	Grade 3	0[a]	0	0	0	1 (1)	0
WBC decrease	Grade 1	2 (1)[a]	0	NA	NA	NA	NA
WBC increase	Grade 1	2 (1)[a]	0	NA	NA	NA	NA
	Grade 2	0[a]	1 (2)	NA	NA	NA	NA
**Post-dose 2**		**MVA-BN-Filo**	**Placebo**	**MVA-BN-Filo**	**Placebo**	**MVA-BN-Filo**	**Placebo**

		12–17 Years		4–11 Years		1–3 Years	
**n**		142	46	143	48	143	48
Chemistry, n (%)							
Alanine Aminotransferase	Any	1 (1)[c]	0	1 (1)[a]	0[d]	2 (1)	2 (4)
Aspartate Aminotransferase	Any	0[c]	0	1 (1)[a]	0[d]	3 (2)	2 (4)
	Grade 3	0[c]	0	1 (1)[a]	0[d]	2 (1)	0
Creatinine	Grade 3	0[c]	0	0[a]	0[d]	1 (1)	0
Haematology							
Haemoglobin	Grade 2	NA	NA	7 (5)	4 (8)[d]	5 (3)	1 (2)
	Grade 3	NA	NA	0	0[d]	6 (4)	0
Haemoglobin change from baseline	Grade 1	82 (58)[c]	27 (59)	NA	NA	NA	NA
	Grade 2	12 (8)[c]	8 (17)	NA	NA	NA	NA
	Grade 3	8 (6)[c]	3 (7)	NA	NA	NA	NA
Platelets	Grade 1	1 (1)[c]	1 (2)	0[a]	0[d]	0	0
	Grade 2	0[c]	0	0[a]	0[d]	4 (3)	0
	Grade 3	0[c]	0	1 (1)[a]	0[d]	0	1 (2)
WBC decrease	Grade 1	1 (1)[c]	1 (2)	NA	NA	NA	NA
WBC increase	Grade 1	2 (1)[c]	0	NA	NA	NA	NA
	Grade 2	1 (1)[c]	0	NA	NA	NA	NA

- ≥12 years: Grading is based on the Food and Drug Administration (FDA) Toxicity Grading Scale for Healthy Adult and Adolescent Volunteers Enrolled in Preventive Vaccine Clinical Trials
- <12 years: Grading is based on the Division of Microbiology and Infectious Diseases (DMID) Toxicity Tables For Use in Trials Enrolling Children Greater Than 3 Months of Age.
- A toxicity grade is considered as following vaccination in a period and/or phase if it is worse than the corresponding baseline record.
- Alanine aminotransferase, aspartate aminotransferase and creatinine refer to increases in values, whereas haemoglobin change from baseline, lymphocytes and platelets refer to decreases in values.

This table only includes laboratory abnormalities that were reported between dose 1 vaccination and 28-days post-dose 1, and between dose 2 vaccination and 28-days-post-dose 2.

n=total number of participants with data; n (%)=number (percentage) of participants with 1 or more events, where the denominator is the number of participants with available reactogenicity data after the given dose; NA=not applicable; WBC=white blood cell.
[a]n=142; [b]n=143; [c]n=141; [d]n=47.

2.7. ***Table S7*: Geometric mean (with 95% CI) EBOV GP-specific binding antibody concentrations before and after vaccination and the responder rate in study participants**

	**12–17 years**		**4–11 years**		**1–3 years**	
	**Ad26.ZEBOV, MVA-BN-Filo**	**MenACWY, Placebo**	**Ad26.ZEBOV, MVA-BN-Filo**	**MenACWY, Placebo**	**Ad26.ZEBOV, MVA-BN-Filo**	**MenACWY, Placebo**
Day 1 (Baseline)						
n	142	46	130	43	123	41
GMC (95% CI)	65 (52–81)	72 (46–111)	62 (49–78)	39 (<LLOQ–54)	<LLOQ (<LLOQ–<LLOQ)	<LLOQ (<LLOQ–<LLOQ)
Day 57 (56-days post-dose 1)						
n	142	46	133	44	125	41
GMC (95% CI)	314 (269–366)	78 (51–119)	390 (334–456)	47 (<LLOQ–67)	693 (591–812)	<LLOQ (<LLOQ–<LLOQ)
Responder (n/N* (%))	91/142 (64)	3/46 (7)	92/129 (71)	3/43 (7)	115/122 (94)	1/41 (2)
(95% CI)	(56–72)	(1–18)	(63–79)	(2–19)	(89–98)	(0–13)
Day 78 (21-days post-dose 2)						
n	134	46	124	43	124	38
GMC (95% CI)	9929 (8172–12064)	74 (48–114)	10212 (8419–12388)	42 (<LLOQ–60)	22568 (18426–27642)	<LLOQ (<LLOQ–38)
Responder (n/N* (%))	131/134 (98)	1/46 (2)	119/120 (99)	3/42 (7)	118/121 (98)	1/38 (3)
(95% CI)	(94–100)	(1–12)	(95–100)	(2–20)	(93–100)	(0–14)
Day 240 (179-days post-dose 2)						
n	135	44	126	41	122	38
GMC (95% CI)	469 (397–554)	79 (51–123)	442 (377–518)	42 (<LLOQ–67)	713 (598–849)	<LLOQ (<LLOQ–39)
Responder (n/N* (%))	99/135 (73)	3/44 (7)	90/122 (74)	3/40 (8)	111/119 (93)	4/38 (11)
(95% CI)	(65–81)	(1–19)	(65–81)	(2–20)	(87–97)	(3–25)
Day 360 (359-days post-dose 1)						
n	132	43	123	41	120	39
GMC (95% CI)	386 (326–457)	93 (55–157)	436 (375–506)	46 (<LLOQ–72)	750 (629–894)	<LLOQ (<LLOQ–42)
Responder (n/N* (%))	92/132 (70)	6/43 (14)	85/119 (71)	5/39 (13)	112/117 (96)	4/39 (10)
(95% CI)	(61–77)	(5–28)	(62– 79)	(4–27)	(90–99)	(3–24)

A participant was a responder at a considered time-point if the sample interpretation was negative at baseline and positive post baseline and the post-baseline value was greater than 2.5x LLOQ, or sample interpretation was positive both at baseline and post baseline and there was a greater than 2.5-fold increase from baseline.

The geometric mean concentration and corresponding confidence interval are shown on the reported scale (ELISA units/mL). Exact Clopper-Pearson confidence interval is shown for the corresponding responder rate.

Vaccines: Ad26.ZEBOV at a dose of $5x10^{10}$ vp; MVA-BN-Filo at a dose of $1x10^{8}$ Inf.U. Control: Meningococcal quadrivalent (serogroups A, C, W135 and Y) conjugate vaccine (MenACWY; dose 1), Placebo (dose 2). n=number of participants with data; CI= confidence interval; GMC=geometric mean concentration; LLOQ=lower limit of quantification;
N*=number of participants with data at baseline and at that time point.

2.8. ***Table S8*: Geometric mean (with 95% CI) EBOV GP-specific neutralizing antibody concentrations before and after vaccination and the responder rate in study participants**

	**12–17 Years**		**4–11 Years**		**1–3 Years**	
	**Ad26.ZEBOV, MVA-BN-Filo**	**MenACWY, Placebo**	**Ad26.ZEBOV, MVA-BN-Filo**	**MenACWY, Placebo**	**Ad26.ZEBOV, MVA-BN-Filo**	**MenACWY, Placebo**
Day 1 (Baseline)						
n	41	13	41	14	38	18
GMT (95% CI)	<LLOQ	<LLOQ	<LLOQ	<LLOQ	<LLOQ	<LLOQ
Day 78 (21 days post-dose 2)						
n	35	10	40	13	36	18
GMT (95% CI)	2120 (1444–3111)	<LLOQ	2483 (1719–3587)	<LLOQ	8142 (4869–13615)	<LLOQ (<LLOQ–149)
Responder (n/N* (%))	33/35 (94)	0/10 (0)	38/40 (95)	0/13 (0)	34/36 (94)	1/18 (6)
(95% CI)	(81–99)	(0–31)	(83–99)	(0–25%)	(81–99)	(0–27)
Day 360 (359 days post-dose 1)						
n	40	14	41	14	37	16
GMT (95% CI)	<LLOQ (<LLOQ–<LLOQ)	<LLOQ	<LLOQ (<LLOQ–126)	<LLOQ (<LLOQ–LLOQ)	252 (189–336)	<LLOQ
Responder (n/N* (%))	3/40 (8)	0/13 (0)	6/40 (15)	1/14 (7)	18/37 (49)	0/16 (0)
(95% CI)	(2–20)	(0–25)	(6–30)	(0–34)	(32–66)	(0–21)

Vaccines: Ad26.ZEBOV; MVA-BN-Filo. Control: Meningococcal quadrivalent (serogroups A, C, W135 and Y) conjugate vaccine (MenACWY; dose 1), Placebo (dose 2).
CI=confidence interval; GMC=geometric mean concentration; LLOQ=lower limit of quantification;n=number of participants with data; N*=number of participants with data at baseline and at that time point.
GMTs and their corresponding CIs are shown on the reported scale (psVNA IC50 titer). For the responder rates, Exact Clopper-Pearson Cis are shown. A participant was a responder at a considered time point either (i) if the sample interpretation was negative at baseline and positive post baseline and the post baseline value was greater than 2x LLOQ; or (ii) if the sample interpretation was positive both at baseline and post baseline and there was a greater than 2-fold increase from baseline.

2.8. ***Table S9*: Ad26 neutralising antibodies (Ad26 VNA, IC_{90} titre): Geometric mean titre and sample interpretation in study participants per protocol analysis set**

	12–17 years		4–11 years		1–3 years	
	**Ad26.ZEBOV, MVA-BN-Filo**	**MenACWY, Placebo**	**Ad26.ZEBOV, MVA-BN-Filo**	**MenACWY, Placebo**	**Ad26.ZEBOV, MVA-BN-Filo**	**MenACWY, Placebo**
Day 1 (Baseline)						
n	142	46	134	45	124	40
GMT (95% CI)	77 (58–101)	65 (39–109)	143 (101–201)	124 (73–212)	19 (<LLOQ–26)	22 (<LLOQ–41)
Positive sample (n (%))	111 (78)	32 (70)	103 (77)	35 (78)	25 (20)	10 (25)
(95% CI)	(71–85)	(54–82)	(69–84)	(63–89)	(14–28)	(13–41)

The geometric mean titre and corresponding confidence interval are shown on the reported scale (IC_{90} titer).
Exact Clopper-Pearson confidence interval is shown for the corresponding sample interpretation rate.
Vaccines: Ad26.ZEBOV at a dose of $5x10^{10}$ vp; MVA: MVA-BN-Filo at a dose of $1x10^{8}$ Inf.U. Control: Meningococcal quadrivalent (serogroups A, C, W135 and Y) conjugate vaccine (MenACWY; dose 1), Placebo (dose 2). n=number of participants with data. CI=confidence interval; GMT=geometric mean titre; LLOQ=lower limit of quantification.

2.9. *Figure S1*: Correlation between EBOV GP-specific binding antibody concentrations and EBOV GP-specific neutralising antibody titres in study participants

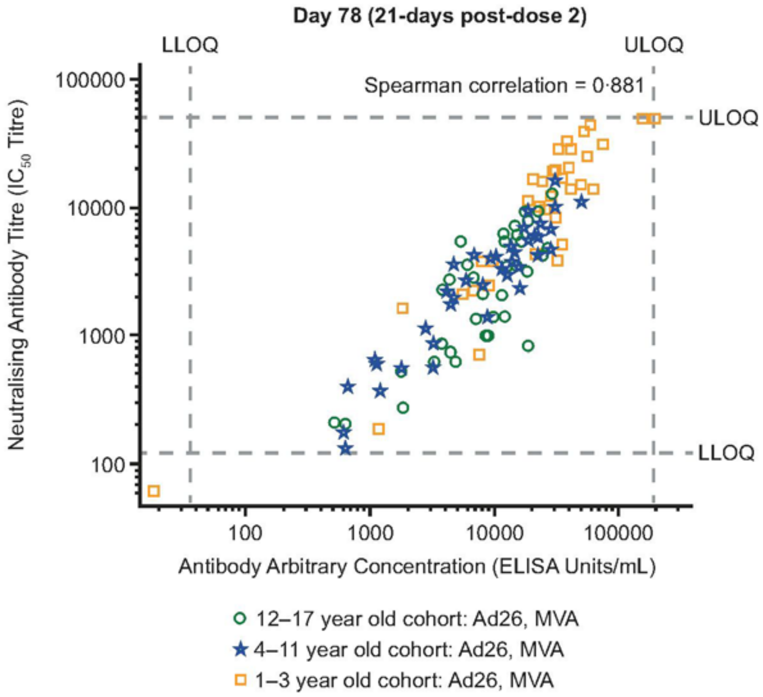

Ad26: Ad26.ZEBOV at a dose of $5x10^{10}$ vp; MVA: MVA-BN-Filo at a dose of $1x10^{8}$ Inf.U.
LLOQ=lower limit of quantification; ULOQ=upper limit of quantification
Control participants are excluded from this figure.
The partial Spearman correlation coefficient, controlling for age group, is shown.

2.10. *Figure S2*: Correlation between pre-vaccination Ad26 neutralising antibody titres and EBOV GP-specific binding antibody concentrations in study participants

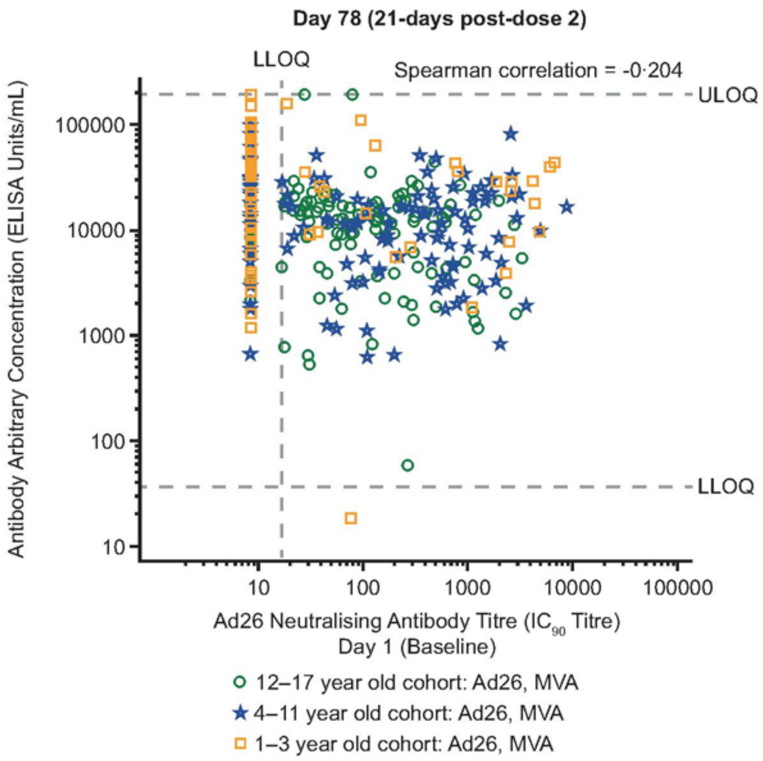

Ad26: Ad26.ZEBOV at a dose of $5x10^{10}$ vp; MVA: MVA-BN-Filo at a dose of $1x10^{8}$ Inf.U.
LLOQ=lower limit of quantification; ULOQ=upper limit of quantification
Control participants are excluded from this display.
The partial Spearman correlation coefficient, controlling for age group, is shown.

2.11. *Figure S3*: Correlation between EBOV GP-specific binding antibody concentrations at baseline and post-vaccination in study participants

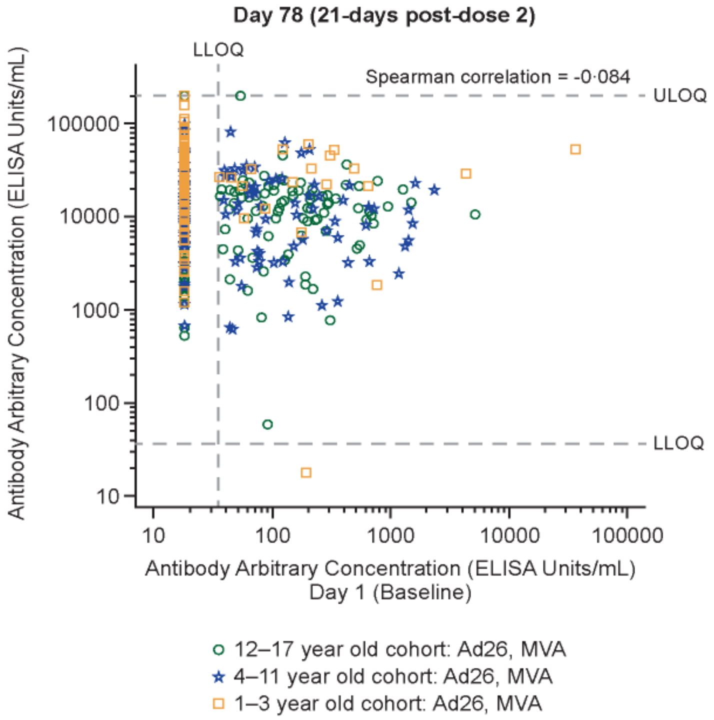

Ad26: Ad26.ZEBOV at a dose of $5x10^{10}$ vp; MVA: MVA-BN-Filo at a dose of $1x10^{8}$ Inf.U.
LLOQ=lower limit of quantification; ULOQ=upper limit of quantification
Control participants are excluded from this display.
The partial Spearman correlation coefficient, controlling for age group, is shown.

3. EBL3001 STUDY GROUP

List of contributors to the study over the four-year study duration period (2015–2019):

Biometrics team

Manager: Moughrphillea Kargbo. Operators: Elizabeth Bockarie, Ndeayah L James, Aminata Kabbah, Aminata Kamara, Kabba H Koroma, Samuel O Langley, Nathaniel William. Technician: Randolph Kessebeh.

Communications and community liaison

Communications Manager: Thomas Mooney. Communications Specialist (Ministry of Health and Sanitation): Lansana Conteh. Community Engagement Coordinator: Elizabeth Smout. Community Liaison Assistants: Kadie Allieu, Kadiatu Bangura, Masian S Bangura, Mohamed A Bangura, Hassan Jalloh, Abu Bakarr Jalloh, Isata Kamara, Mohamed Kamara, Adama Konteh, Suad Koroma, Claudia Marrah, Mohamed Sesay, Mohamed Tejan Sesay. Community Liaison Officer: Abdul Tejan Deen.

Community health officers

AbuBakarr Jalloh, Richard M Kaimbay, Desmond Kain, Abdul Kamara, Edward L Kamara, Mohamed Potho Kamara, Osman J Kamara, Ishmael Kamara, Stephen LM Kamara, Mohamed Kanneh, Alhassan H Koroma, Dauda Lahai, Ibrahim S Mansaray, Woria S Marah, Mary J Massaquoi, Abubakarr Nabie, Nyayia S Saidu, Ishmael Samai, Joseph N Tengheh, Andrew S Turay.

Data management

Assistant Data Manager: Alpha Fornah, Francis Sesay, Alphajor Sow, Ernest Swaray, Foday Mansaray. Data clerks: Theodore Ade-Cole, Lamin M Bangura, Mohamed L Conteh, Aminata Kabbah, Augustine M Koroma, Mohamed Koroma, Augustine Sam, Thelma Scott, TutuSessie, Joe-Henry C Sunders, Sorie I-S Turay, James Weekes. Data Manager: Mahmud Sheku. Senior Data Manager: Lorna Gibson (interim), Dickens Kowuor

IT manager: Irrfan Ahamed.

Field workers

Field supervisors: William Allieu, Divine U Kabba, Francess J Kamara, Mohamed S Kebbie, Momoh Pessima, Abdulai Wurie. Field workers: Fatmata Bah, Abdulai I Bangura, Richard AS Bangura, Lovetta Blango, Sia Boima, Memunatu Conteh, Yusufu Conteh, Mohamed L Daramy, Osman Fofanah, Eleanor George, Thelma F Hanson, Mariama I Jalloh, Mathew Kalawa, Abdulai M Kamara, Francis E Kamara, Gibril M Kamara, Hassanatu M Kamara, Posseh BD Kamara, Ramatu T Kamara, Rugiatu Kamara, Derick P Kanneh, Moses Kanneh, Ishmael Komeh, Musu Koroma, Maseray Kuyateh, Fatmata F Mansaray, Marina M Mansaray, Akim B Sillah, Musa A Tarawally, Osman S Turya, James B Yawmah.

Investigators (principals and directors)

Principal Investigator (COMAHS): Bailah Leigh. Principal Investigators; LSHTM: Deborah Watson-Jones, Brian Greenwood. Investigator /COMAHS Scientific Director: Mohamed H Samai. Investigator / COMAHS Clinical Director: Gibrilla Fadlu Deen. Investigator / EPI Director: Dennis Marke. Tom Sesay

LSHTM Senior Team: Peter Piot, Peter Smith, John Edmunds, Shelley Lees, Heidi Larson, Helen Weiss, Patrick Wilson

Investigators and site coordination and medical team (site)

Assistant Trial Coordinator: Robert Phillips. Epidemiologist: Caroline Maxwell. Investigator / Trial Manager: David Ishola. Investigators / Trial Coordinators: Muhammed Afolabi, Frank Baiden. Trial Coordinators: Pauline Akoo, Kwabena Owusu-Kyei, Daniel Tindanbil. Trial Manager: Hilary Bower (interim), James Stuart (interim). Investigators / Senior study doctors: Osman M Bah, Baimba T Rogers, Alimamy Serry-Bangura, Ibrahim Bob Swaray. Investigators / Study doctors: Agnes Bangura, Ifeolu John David, David GM Davies, Joseph Alpha Kallon, Abu Bakarr Kamara, Ibrahim F Kamara, Michael M Kamara, Foday E Morovia, Foday B Suma, Frederick Thompson. Kambia District Medical Officers: Mariama Murray, Foday Sesay. Kambia Government Hospital Medical Superintendents: Osman Kakay, Foday Suma, Isaac Sesay.

Coordination and project management team (London)

Assistant Trial Coordinator: Julie Foster, Robert Phillips. Trial Coordinators: Daniela Manno, Kate Gallagher, Sharon Cox (interim), Natasha Howard (interim). Project Assistant: Maria Cesay, Paola Torrani, Sarah Sharma. Project managers: Emily Snowden, Thom Banks, Tomas Harber, Jennifer Brown, Kelly Howard, Natalie Melton. Senior Project Managers: Stuart Malcolm, Suzanne Welsh.

Mathematical modelling team (London)

Rosalind Eggo, Mario Jendrossek, Carl Pearson.

Janssen team

Kim Offergeld, Clinical Program Leader, Director, Global Clinical Operations. Camille FERRAULT, Clinical Project Management Lead. Meilssa Van Alst, GCDO. Navdeep Mahajan, GTM. Marleen Van Looveren, Medical writer. Sylvia Van Ballaert, Medical writer. Tinne De Cnodder, Data manager. Nico Grobler, Data manager. Len Roza, Quality Manager. Tammy Liberi, Independent Drug Monitoring Manager. Lee Armishaw, Senior Programmer. Chantal VERKLEIJ, EMEA Regulatory Liaison. Tracy HENRICK, Stat Prog Leader. Agnieszka Banaszkiewicz, Clinical Trial Assistant.

Laboratory team

Laboratory Director: Brett Lowe. Laboratory Manager: Ken Awuondo. Laboratory Scientists: Hannah Hafezi, Emma Hancox, Brian Kohn, Godfrey O Tuda. Laboratory Technicians: Fatmata Koroma, Alima Kamara, Gerald Bangura, Mattu T Kroma, Lamin Fofanah, Alfred Pessima, Maariam Rogers, Osman Sheriff. Phlebotomists / Laboratory Assistants: Tosin W Ajala, Joseph Fangawa, Sahr Foday Jr, Issa Jabbie, Bobson Mansaray, Haja A Mansaray, Kandeh Sesay.

MOTECH (mobile technology) team

Analysts: Millicent K Charles, Pamela C Heroe, Mohamed Lamin Karbo, Ibrahim S Yansaneh. Administrators: Seth Gogo Egoeh, Augustin Trye. Consultant: Monica Amponsah.

Monitoring team

ICON Government and Public Health Solutions: Lori Donelson, Therese Sylvester, Victorine Owira, Gonzaga Onyuka, Lilian Nambuchi, Amos Oburu, Duncan Apollo, Lahai Vandi

Nurses

Nurses: Nasiru D Alghali, Adama Bah, Isata J Bangura, Alrine Cheryl Cole, Saidu Fofanah, Hawa Umu Jalloh, Kadijatu FN Jalloh, Nini Jalloh, Haja U Kabba, Jennifer N Kabba, Marion Kabba, Rashidatu Kamara, Juliana S Kamara, Finda Kanjie, Aminata P Kanu, Iynnah Kargbo, Gladys Kassa-Koroma, Susanette B Koroma, Adikalie Sankoh, Theresa Sankoh, Olaimatu D Sesay, Hellen Wilhem, Cecilia T Williams. Nurse assistants: Isata Bangura, Yeama Ben-Rogers, Adama Jalloh, Fatmata J Jamboria, Nashiratu Kamara, Isha Kanawah, Adama T Kargbo, Isata Swaray. Senior nurses: Lucinda Amara, Isatu Bundu, Hannah B Jakema, Kadiatu Kamara, Millicent F Sheku.

Paediatrics team

Paediatricians: Qadri Adeleye, Irene Akhigbe, Respicious Bakalemwa, Neema P Chami, Tulla Sylvester. Paediatric nurses: Lisa Altmann, Bomposseh Kamara.

Pharmacy / vaccine cold chain team

Cold Chain Coordinator Specialist: Karel van Roey. Cold Chain Officers: Philip Conteh, Moses Samura, Victor Gandie, Mohamed Marrah, Emmanuel Moinina, Joseph Kalokoh. District Pharmacist: Mohamed I Bangura. Lead Pharmacists: Samuel Bosompem, Trudi Hilton (interim), Morrison O Jusu. Pharmacists: Paul Borboh, Augustine S Brima, Amanda FY Caulker, Ahamed Kallon, Bockarie Koroma, Rachel C Macauley, Tamba MD Saquee, Harold I Williams.

Project management group

Administrators: Abdul Rahman Bangura, Juliana Fornah, Bockarie Idriss. Finance Accountant: Marion Sillah. Finance Director: Waltina Mackay. Finance Managers: Belayneh Aleghen, Tamba Murray. HR Director: Joseph Edem-Hotah. IT manager: Tamba Fatorma. Logistics Managers: Frederick Amara, Mohamed Kamara. Logistics Officers: Santigie Bangura, Elba Bonnie, Musa Sannoh. Operations Manager: Alexandra Donaldson, Samuel Ndingi, Dennis Nyaberi, Martin Pereira (interim), Antony Rothwell. Programme Manager: Vanessa Vy. Senior Administrator: Leonard Nyallay.

Quality Assurance officials

Quality Administrators: Augustin Fombah, Samuel Saidu. QA Manager: Emma Hancox.

Registration team

Clerks: Thomas P Dambo, Philip J Fakaba, Mary ME Fatorma, Rexmonda H Freeman, Cordelia L Johnson, Memunatu Kamara, Darlinda B Kogba, Alfred Lahai, Willieta Vincent, Nabieu Yambasu.

Social science team

Graduate Researchers: Mahmood Bangura, Angus Tengbeh. Research Assistants: Kadiatu Bangura, Rosetta Kabia. Transcriber: Alhaji M Nyakoi. Social Science Leads: Mike Callaghan, Luisa Enria, Shona Lee.

4. STUDY PROTOCOL
